# Supplementary material for: Men's and women's experiences of violence and traumatic events in rural Côte d'Ivoire before, during and after a period of armed conflict
Source: BMJ Open. 2014 Feb 25;4(2):e003644. doi: 10.1136/bmjopen-2013-003644 (PMC3939650; doi:10.1136/bmjopen-2013-003644)
Supplement: Author's manuscript [file bmjopen-2013-003644.draft_revisions.pdf]

Sexual and physical violence in a West-African conflict-affected setting: Men's and women's experiences of violence and traumatic events in Côte d'Ivoire before, during and after a period of armed conflict

Journal:	*BMJ Open*
Manuscript ID:	bmjopen-2013-003644
Article Type:	Research
Date Submitted by the Author:	23-Jul-2013
Complete List of Authors:	Hossain, Mazeda; London School of Hygiene & Tropical Medicine,<br>Zimmerman, Cathy; London School of Hygiene & Tropical Medicine,<br>Kiss, Ligia; London School of Hygiene & Tropical Medicine,<br>Kone, Drissa; International Rescue Committee,<br>Topolska, Monika; International Rescue Committee,<br>Manan, David; International Rescue Committee,<br>Lehman, Heidi; International Rescue Committee,<br>Watts, Charlotte; London School of Hygiene & Tropical Medicine,
**Primary Subject Heading**:	Global health
Secondary Subject Heading:	Global health, Public health
Keywords:	Violence, Armed conflict, war, Intimate Partner Violence

SCHOLARONE™
Manuscripts

For peer review only

Sexual and physical violence in a West-African conflict-affected setting:

Men's and women's experiences of violence and traumatic events in Côte d'Ivoire before, during and after a period of armed conflict

For peer review only

Mazeda Hossain, MSc[1]
Cathy Zimmerman, PhD[1]
Ligia Kiss, PhD[1]
Drissa Kone[2]
Monika Topolska[2]
David Manan, MA[2]
Heidi Lehman, MA[2]
Charlotte Watts, PhD[1]

[1] London School of Hygiene & Tropical Medicine
[2] The International Rescue Committee (IRC)

Corresponding author:
Mazeda Hossain
Department of Global Health & Development
London School of Hygiene & Tropical Medicine
15-17 Tavistock Place
London
WC1H 9SH
United Kingdom
Email: Mazeda.Hossain@lshtm.ac.uk

For peer review only - http://bmjopen.bmj.com/site/about/guidelines.xhtml

ABSTRACT

Objective
Sexual violence in conflict is recognized as a crime against humanity yet there is limited population data on its magnitude in different settings and little on the prevalence of other serious forms of violence. Therefore, we measured women's and men's experiences of gender based violence and other traumatic events within a West African conflict-affected setting before, during, and after a period of active armed conflict (2000-2007).

Design
Cross-sectional, household survey.

Setting
12 rural communities directly impacted by the Crisis in Côte d'Ivoire, spanning regions controlled by government forces, rebels, and UN peacekeepers in 2008.

Participants
2,678 women and men aged 15 to 49.

Primary outcome measures
Violence exposures measured since age 15. Questions included intimate partner physical and sexual violence; physical and sexual violence by others (including combatants) and exposure to traumatic events before, during and after the Crisis period (2000-2007).

Results
Physical and/or sexual violence since age 15 was reported by 57.1% women and 40.2% men (p=0.01); 29.9% women and 12.3% men reported past year exposure. Nearly one in ten women (9.9%) were forced to have sex by a non-partner since age 15, and 14.8% reporting their first sexual experience was forced. Combatants were rarely reported as sexual violence perpetrators (0.3% women). Sexual violence against men was lower (5.9% since age 15, 0.1% in past year). After the Crisis, intimate partner physical violence was the most frequently reported form of violence and highest among women (20.9% women, 9.9% men, p=0.00).

Conclusions
Sexual violence in conflict remains a critical international policy concern. However, in many conflict settings, other forms of violence, including sexual and physical partner violence, are more widespread. Alongside service provision for rape survivors, our findings underscore the need for post-conflict reconstruction efforts to invest in programs to prevent and respond to partner violence and trauma.

For peer review only

For peer review only - http://bmjopen.bmj.com/site/about/guidelines.xhtml

Summary

Article focus

- This study suggests a very different picture from current international policy priorities, which predominantly focuses on conflict-related sexual violence.
- We sought to address the limited population-level data on sexual and other forms of gender based violence experienced before, during and after an armed conflict period in a West African setting.
- We assessed the differences in the types and severity of violence experienced by women and men.

Key messages

- Violence experiences over a lifetime were common. More than half of all women (57%) and 40% of men, reported experiencing physical or sexual violence. A third of women (30%) continued to experience violence in the 12 months after the Crisis period.
- International attention to conflict-related sexual violence is warranted however, in our study, women experienced the highest levels of violence both within and outside of their homes, and were most likely to report the most severe forms of physical violence by an intimate partner (i.e. dragged, kicked, choked) rather than a combatant, in addition to experiencing sexual violence by partners and non-partners. Family and acquaintances were reported as perpetrators more often than combatants.
- Although women reported violence in greater proportions, reconstruction efforts should not ignore men who report high levels of non-partner physical violence. Men are also victims of multiple forms of violence (including sexual violence) and traumas and may be less likely to disclose and receive support.

Strengths and limitations

- This study presents the first temporal violence and trauma prevalence data from regions spanning rebel, government and UN-controlled forces in Côte d'Ivoire.
- This study was not nationally representative however when compared to regional DHS data, the study sample is regionally representative.
- Sexual violence figures should be interpreted with caution as we did not explore the broader range of sexual abuse and response bias is possible.

For peer review only

For peer review only - http://bmjopen.bmj.com/site/about/guidelines.xhtml

INTRODUCTION

The past decade has seen unprecedented recognition of sexual violence in conflict.[1] The UN Security Council alone has issued nine resolutions focused on sexual violence (SV) in conflict and fragile state settings since 2000.[2] Assuming the G8 presidency in 2013, the UK announced its firm commitment to address violence against women and girls and in April launched the *G8 Declaration on Preventing Sexual Violence.* This declaration comes with a commitment of £23 million by the G8 nations. In the same year, the UK Foreign Office announced the *Preventing Sexual Violence Initiative,*[3] towards which the UK Government dedicated £10 million to end sexual violence in conflict.[4]

Yet, the limited evidence on the prevalence and patterns of violence in conflict-affected settings makes it difficult for governments, humanitarian and donor agencies to determine how to target their resources most effectively.[5]

Not surprisingly, robust national level data on the extent of SV are extremely difficult to compile, with current prevalence estimates ranging widely. For example, in the Democratic Republic of Congo (DRC), reports on the extent of conflict-related sexual violence range from 17·8%-39·7% among women and 4%-23.6% among men, due, in part, to methodological differences.[6-8] In the same setting, women also report high levels of violence by an intimate partner (termed intimate partner violence (IPV) or domestic violence), with 35·3% of ever-partnered women reporting sexual partner violence and 56·9% reporting physical partner violence.[9] More recently, data has emerged from Liberia showing high levels of violence and trauma especially among women.[10]

Such data have led to the growing recognition that rape in war is one of numerous forms of violence in conflict-affected settings. Sexual and physical IPV, child sexual abuse, forced marriage, sexual harassment, and rape by non-combatants are also of major concern.[11-14] For example, the most recent Human Security Report (2012) highlighted the discrepancy between the evidence and the international focus on sexual violence in conflict, citing in particular evidence that domestic sexual violence may be more prevalent than rape.[15 12, 16, 17]

Côte d'Ivoire is a West African country that has experienced a protracted conflict, known as *the Crisis*, since a coup d'état in 1999.[18-20] In 2002, a UN-French controlled buffer zone was created, effectively dividing the nation into a rebel controlled in the north and government controlled south.[21] The first steps of a peace agreement were brokered in 2007 which was followed by a year with limited conflict-related violence in between 2007 and 2008. In 2008 however, the country again entered a period of instability before transitioning to an elected President in 2011.[22] Côte d'Ivoire once considered the 'jewel of West Africa' remains a critical country for regional West African security as it maintains deep ties to neighbouring countries (Mali, Burkina Faso, Ghana, Guinea, Liberia) and other West African nations (Togo, Benin, Sierra Leone, Niger) through migration, trade and remittances. The impact of over a decade of instability and violence is still not known.[23] As the country transitions to the post-conflict period, an understanding of the types of violence and trauma exposures in Côte d'Ivoire may provide insights into programming for health, legal and social sectors both within the country and in neighbouring countries such as Mali who are currently experiencing similar low-level ethnic tensions.[24]

This paper presents the findings from a household survey on women's and men's exposures to interpersonal violence and trauma in 12 rural villages across 6 administrative districts in Côte d'Ivoire, prior to (pre-1999), during (2000-2007) and one year after a period of active conflict (2007-08).

For peer review only - http://bmjopen.bmj.com/site/about/guidelines.xhtml

METHODS

Study design and sample

A cross-sectional community survey was conducted in Nov-Dec 2008 among 2,684 respondents (53% women, 47% men) aged 15-49 years in 12 rural communities across six administrative districts in Côte d'Ivoire. These regions included: Yamoussoukro, Daloa, Bouaflé, Bangolo, Danané, and Duekoué. This survey was carried out as part of a formative assessment for a cluster randomised trial to evaluate the impact of an IPV prevention intervention implemented by a humanitarian organisation in Côte d'Ivoire. The findings were used to inform the intervention design, which was implemented between 2010-2012 in the same communities.

The study communities were purposively selected based on their accessibility and current relationship with the humanitarian organisation, the International Rescue Committee (IRC). All communities had similar socio-economic profiles, with residents relying primarily on agriculture as their main income source. The administrative districts spanned regions controlled by the government, rebels, or UN peacekeepers.

Within each community, a representative sample was obtained by first mapping all households to create a sampling list of individuals. All households within each community were eligible to participate. Due to ethical and safety concerns related to disclosure, we did not aim to interview male and female respondents in the same household. Instead, half of the households in the community were randomly allocated to be 'male' respondent households, and the remainder to be 'female' respondent households. In each household, all eligible household members of the same sex who met the eligibility criteria (15-49 years old and resident in the community for at least one year) were invited to participate and be interviewed in private by an interviewer of the same sex. The mapping found that 12,041 individuals lived in the 12 study communities, of which 3,471 were eligible to participate and 2,869 adults completed an interview (83% response rate). Non-response was generally attributed to being out of town, illness or work reasons.

Violence & trauma measures

Behaviourally specific questions were used to ask respondents about their experiences of various acts of violence perpetrated either by an intimate partner (termed 'intimate partner violence') or by other perpetrators, such as neighbours, relatives, teachers and combatants (termed 'non-partner violence').

The IPV module drew on survey instruments used internationally to study IPV.[25-27] All ever-partnered participants were asked: "Has your partner ever..." perpetrated a specific act of violence, and if so when (last 12 months, before the last 12 months) and how often (never, sometimes, often) for each time period. Physical violence acts included being (1) slapped, pushed or (2) hit with something that could hurt you. Severe physical acts were measured by affirmative reports of being (1) kicked, dragged, beaten, (2) choked, burned or (3) threatened with a weapon. Women were also asked about experiences of partner SV, and specifically were asked whether they had been (1) physically forced to have sex; or (2) forced to have sex due to fear.

An individual was considered to have experienced physical partner violence if she/he reported more than one experience of the following acts: hit with a fist or something else, slapped or had something that could hurt thrown at her/him, pushed or shoved. Severe physical violence was defined as reporting at least one experience of the following severe physical violence acts: kicked or dragged, choked or burnt, threatened with a weapon. This categorisation of partner physical violence reflects not only the severity of the act (e.g. the difference between being slapped vs. choked) but also the frequency that the act occurred. It also reflects a more conservative approach that eliminates individuals (women and men) from the overall prevalence who have experienced a

For peer review only - http://bmjopen.bmj.com/site/about/guidelines.xhtml

single act of violence, such as a slap. A woman was considered to have experienced sexual partner violence if she reported one or more experiences of forced sex.

Experiences of non-partner violence measured physical violence as an adult (>15 years old) and sexual violence. For physical violence, questions included: "Since the age of 15, apart from your partner, has anyone ever physically hurt you?" Sexual violence was measured by: "Since the age of 15, apart from your partner, has anyone ever forced you to have sex against your will?" For both types, follow-up questions were asked about the perpetrator, and the timing of the assault in relation to the Crisis.

Traumatic events were measured by drawing on the seven domains designated by the Harvard Trauma Questionnaire as common experiences among war-affected populations.[28] Questions were modified to reflect the traumatic experiences potentially associated with conflict-related violence in Côte d'Ivoire. All participants were asked if they had experienced specific events including: feared for your life, village attacked, witnessed family members seriously hurt/killed, forced to work for someone who attacked your village, forced to have sex with someone who attacked your village, forced to flee your village, family member threatened, seriously hurt by an act of violence, forced to use a weapon against someone, and seriously hurt someone. A binary variable was created to capture participants who experiences above median number of events (5 or more).

In each case respondents who reported affirmatively were asked about the timing: in the last 12 months (2007-2008) [After the Crisis], during the Crisis period (1999-2007) [During the Crisis], or before the coup d'état (pre-1999) [Before the Crisis].

Translation, ethics & data collection

The questionnaire was developed in English and French and then translated and back-translated into eight local Ivorian languages. An intensive group translation method was developed by the research team where local language speakers translated questions individually and then met as a group (5-10 people) to reach a consensus on the local language interpretation. This interpretation was then checked with the study team and other language groups to ensure that the appropriate and similar meaning was captured across the multiple translations. The final instrument underwent another round of pilot testing and further revision before implementation.

Strict ethical procedures were adopted that recognised possible trauma experienced by the study population and the possibility of renewed violence in the study communities or against field staff. To ensure the safety of all participants and researchers, all interviewers participated in an intensive three-week training which included ethical and safety training; and all participants were provided access to psychological and medical support.

Face-to-face interviews were conducted in French or local language in a private setting to reduce levels of bias and improve disclosure. Prior to the start of the interviews, consent for the research project was obtained from village leaders, household heads, and individual participants. Quality control measures included the use of multiple checks during the data collection phase and later double-data entry procedures by the data entry team. Ethical clearance for the project was obtained from the LSHTM in 2008.

Statistical analysis

The data was double-entered and analysis was completed using Stata 12.[29] Descriptive data analysis was performed using the Stata survey module. Final analysis was conducted among completed questionnaires. Prevalence data and 95% confidence intervals were calculated using survey commands to account for clustering at the village level. Bivariate and sub-group comparisons were

For peer review only - http://bmjopen.bmj.com/site/about/guidelines.xhtml

calculated using the Wald test where $p<0.05$ was considered statistically significant. Weighted Demographic Health Survey data[30] from the same study regions was examined to compare the representativeness of the study population against a nationally-representative population.

For peer review only

For peer review only - http://bmjopen.bmj.com/site/about/guidelines.xhtml

RESULTS

Study population

The majority of study participants were under age 30, with approximately one-quarter of women (23·7%) and men (22·6%) between 15-19 years. (Table 1) Lower literacy levels were reported by women (31%) than men (59%), and less than half of all participants reported basic literacy levels (44%). Most women (87%) said they were in a current relationship with a male partner (i.e. husband, boyfriend), and over half were co-habiting (63·6%). Most men (77%) also reported having a current relationship with either a wife or girlfriend, with half (51.8%) living with their female partner. Almost a third (29%) of partnered women and 12% of partnered men reported their relationship was polygamous, in which the male partner had more than one concurrent wife. (Table 1) Comparisons with the 2005 DHS data suggests that the study population surveyed are representative of the regional population, with similar age breakdowns, levels of educational attainment, number of children and percentage of population living in conflict-affected zones. Differences between partnership status and religion are likely attributable to different definitions between the surveys (Table 1).

<< Table 1 >>

Prevalence of sexual and physical violence exposures

More than half of all women (57·1%) and over a third of all men (40·2%) reported an experience of physical and/or sexual violence since age 15. (Table 2) Approximately one-third of women (29·9%) and 12·3% of men reported physical and/or sexual violence in the 12 month period following the Crisis. The reported levels of physical and/or sexual violence by non-partners were very similar between men and women, with 27·7% of women and 29·9% of men reporting violence by a non-partner since age 15, and 3·0% and 3·6% of women and men respectively reporting abuse in the past year. This highlights that the main factor explaining the difference in the overall levels of violence exposure between the sexes can be attributed to the differing levels of IPV experienced by women than men.

<< Table 2 >>

Almost one in ten women (9·9%) reported being forced to have sex by someone other than their partner since age 15, with 1·1% reporting non-partner SV in the past year. For men, the figures were lower but not negligible (5·9% since age 15, 0·1% in the past year). Many women also reported forced sex by a partner, with 29·1% and 14·9% of ever partnered women reporting forced sex ever, and in the past year. (Table 2) In combination, these figures suggest that 32·9% of women have experienced sexual violence since age 15, with most of this sexual violence (24·0% overall) being perpetrated by their intimate partners, and with 5% of women reporting sexual assault by both a partner and other men. Additionally, 14·8% of women and 3·3% of men reported that their first sexual experience was forced (data not shown).

Nearly half of women and over a third of men reported experiencing physical violence since age 15 (47·6% women, 38·0% for men). The levels of physical violence by non-partners were very similar between the sexes over their lifetime (27·7% women, 29·9% men) and in the 12 months following the peace agreement (3·0% women, 3·6% men). In contrast, the reported levels of physical violence by a partner were more than twice as high for women compared to men (20·9% versus 9·9%), with women also being more likely to report experiencing severe acts of physical violence by a partner compared to men (23·9% women, 9·3% men, p=0·00) in their lifetime. (Table 2)

For peer review only - http://bmjopen.bmj.com/site/about/guidelines.xhtml

Perpetrators

Respondents reported a broad range of physical and sexual violence perpetrators. Table 3 shows the prevalence of non-partner sexual and physical violence perpetrators overall, and broken down according to whether the violence occurred before, during, or after the Crisis periods.

Nearly one in ten women (9·9%) reported sexual violence by someone other than their partner, with sexual violence most often perpetrated by male strangers or male acquaintances. Only a small percentage of women reported SV perpetrated by a combatant (0·3%). Fewer men (5·9%) reported sexual violence from someone other than their partner, with the most common perpetrators being female acquaintances (3·4% overall), and female strangers (1·8%). (Table 3)

The reported prevalence of non-partner sexual violence was lower after the Crisis period than during or before the Crisis period, potentially as a result of the difference length of time reflected in each measure. In contrast, the prevalence of SV by an intimate partner remained high (14.9% among women after the Crisis). (Table 2)

Men reported higher levels of experiencing physical violence during the Crisis than women (8·9% women, 12·6% men, p=0·02). The perpetrators typically cited were family members for both women and men, except during the Crisis period, when men were more likely than women to report physical assault from combatants (0·9% women, 4·7% men, p=0·00).

<<Table 3>>

Exposure to traumatic conflict-related events

'*Feared for your life'* was the most commonly traumatic event reported with nearly all participants acknowledging having had at least one experience when they feared for their life since age 15 (90% women, 83% men). As expected, levels of all trauma exposures were higher during the active conflict period. Little difference was noted between women and men, except for fearing for one's life, which was higher among women at all time periods. Among all participants, 19.6% reported experiencing five or more traumatic events in their lifetime. (Figure 1)

<< Figure 1>>

For peer review only - http://bmjopen.bmj.com/site/about/guidelines.xhtml

DISCUSSION

As the international community intensifies its focus on violence against women globally, the UK takes up the G8 Presidency with this same priority, and the stability of the West African region is closely monitored, our findings from Côte d'Ivoire encourage increased attention to violence against women and girls and offer important evidence for policies and programming. Our research provides survey-based data on violence against *both* women and men in a conflict setting. Findings from war-affected contexts show that both sexes are subjected to various forms of abuse. However, our data indicates that when resources are limited, a focus on violence against women and girls (whether through direct services for survivors or prevention efforts) is reasonable at times, as women experience violence in significantly greater proportions and are often exposed to more severe abuses. In our study, women experienced the highest levels of violence both within and outside of their homes, and were most likely to report the most severe forms of physical violence by a partner (i.e. dragged, kicked, choked), in addition to experiencing sexual violence by partners and non-partners.

Our findings also confirm that attention to conflict-related sexual violence is warranted. Yet, at the same time, these results emphasise that focussing narrowly on rape in war in all conflict-affected settings is short-sighted. Our data strongly indicate that violence occurs in many forms and is perpetrated by different individuals, in addition to combatants. The most common perpetrators of violence against women appear to be intimate partners, family members and acquaintances, while men report violence from family members, acquaintances, and during the conflict period, from combatants.

While it is clear that focussing resources specifically on violence against women is well-justified, strategies should not, however, exclude violence experienced by men. Importantly, these findings highlight that men are also victims of multiple forms of abuse, including sexual violence. Furthermore, there is reason to believe that men in conflict settings who have experienced violence, especially sexual violence, are likely to have little support and may be less likely to disclose. Men reported higher levels of non-partner physical violence experiences during and after the Crisis. Non-partner sexual violence was higher among women.

This study was limited as it is not nationally representative of Côte d'Ivoire and covers a sub-section of the country. In addition, we only measured forced sex, did not explore the broader range of forms of sexual abuse that may occur and used survey questions that are widely used among women.[31] Therefore, the sexual violence prevalence figures should be interpreted with caution, as it is unclear how comparable the data is for women and men. Other research suggests that for men, being forced to have sex by a woman may have different implications than for women and is an area that requires further research.[32] There is also the potential for response bias as given the sensitive nature of the questions, participants may be reluctant to report forced sex. Furthermore, although no remuneration was given, there remains the possibility those respondents over-reported, or under-reported, in hopes of receiving services.

Given the range of violence detected in conflict-affected communities, our findings pose a significant challenge to the international community and for the upcoming G8 tenure of the UK government, in particular. To truly make inroads in reducing violence against women, programming must address sexual violence against women *in conjunction with* the many other types of violence that occur to both women and men. Sexual violence, as our data demonstrates, does not occur in isolation. Indeed, especially in contexts where violence is widespread, such as war-torn areas, forms of violence are likely to be interrelated, potentially exacerbating one another.

For peer review only - http://bmjopen.bmj.com/site/about/guidelines.xhtml

Moreover, strategies to address violence occurring in a conflict-affected setting where so many individuals have been exposed to additional traumatic events over the course of their lives, will also need to consider how this range of psychologically damaging circumstances might influence intervention efforts. For example, a majority of study participants reported that they had 'feared for their life', and over 40% of respondents reported being forced to flee their villages due to a violent attack. Promoting recovery and behaviour change (reducing levels of IPV) in a context of fear will undoubtedly be challenging.

Post-conflict reconstruction efforts within West Africa have traditionally focused on security, physical infrastructure and economic development rather than gendered human security issues.[33] However, as Côte d'Ivoire and its neighbour's transition towards peace, the issue of violence against women cannot be ignored. For decision-makers and programmes that have the explicit aim of addressing violence against women in the longer term, this study provides what we hope will be the beginning of a growing evidence-base to foster comprehensive, gender-informed strategies to improve the safety, health and well-being of women, men and children in conflict-affected settings.

For peer review only

For peer review only - http://bmjopen.bmj.com/site/about/guidelines.xhtml

Funding

This work was supported by funding from the NoVo Foundation and the Sigrid Rausing Trust.

Competing Interests

None declared.

Author Contributions

MH, CZ, CW, LK were responsible for the design and conduct of the study. MH, DK were responsible for field management and study instrument development. MT, HL contributed to national level data collection procedures and oversight. All authors contributed to the development and adaptation of the questionnaires and interpretation of the data. MH was responsible for statistical data analysis. MH, CZ, CW drafted the manuscript which was reviewed and approved by all authors. We are grateful to all of the study respondents who participated in the study. We also gratefully acknowledge the dedicated commitment of IRC country staff and the research field staff.

Ethical approval for this study was received from the London School of Hygiene & Tropical Medicine Ethics Committee in 2008.

Data sharing

Preliminary study findings have been presented to the donors, at specialised conferences and several expert group meetings on sexual violence in conflict settings. The data contained in this report is not publically available in a peer-review format.

For peer review only

References

1. Bastick, M., K. Grimm, and R. Kunz, *Sexual Violence in Armed Conflict: Global Overview and Implications for the Security Sector*. 2007, Geneva Centre for the Democratic Control of Armed Forces: Geneva. p. 216.
2. United Nations High Commissioner for Refugees (UNHCR), *Action against Sexual and Gender-Based Violence: An Updated Strategy*. 2011, UNHCR.
3. UK Department for International Development (DfID), *Press release: Helping millions of women and girls access better justice and support against violence*. 2012, DfID: London.
4. UK Foreign & Commonwealth Office and UK Department for International Development. *News Story: UK announces additional funding to address conflict sexual violence*. 2013 [cited; Available from: https://www.gov.uk/government/news/uk-announces-additional-funding-to-address-conflict-sexual-violence.
5. World Health Organization (WHO), *Meeting Report: Sexual Violence in Conflict: Data and Data Collection Methods*. 2008, WHO: Geneva.
6. Casey, S.E., et al., *Care-seeking behavior by survivors of sexual assault in the Democratic Republic of the Congo.* Am J Public Health, 2011. **101**(6): p. 1054-5.
7. Duroch, F., M. McRae, and R.F. Grais, *Description and consequences of sexual violence in Ituri province, Democratic Republic of Congo.* BMC Int Health Hum Rights, 2011. **11**: p. 5.
8. Johnson, K., et al., *Association of sexual violence and human rights violations with physical and mental health in territories of the Eastern Democratic Republic of the Congo.* JAMA, 2010. **304**(5): p. 553-62.
9. Ministère du Plan and Macro International Inc, *Enquête Démographique et de Santé, République Démocratique du Congo 2007*. 2008, Ministère du Plan, Macro International: Calverton, Maryland, USA.
10. Vinck, P. and P.N. Pham, *Association of exposure to intimate-partner physical violence and potentially traumatic war-related events with mental health in Liberia.* Soc Sci Med, 2013. **77**: p. 41-9.
11. Watts, C. and C. Zimmerman, *Violence against women: Global scope and magnitude.* Lancet, 2002. **359**: p. 1232-1237.
12. Geneva Declaration Secretariat, *Global Burden of Armed Violence 2011: Lethal Encounters*. 2011, Cambridge University Press: Cambridge.
13. Peterman, A., T. Palermo, and C. Bredenkamp, *Estimates and determinants of sexual violence against women in the Democratic Republic of Congo.* Am J Public Health, 2011. **101**(6): p. 1060-7.
14. Bartels, S.A., et al., *Sexual violence trends between 2004 and 2008 in South Kivu, Democratic Republic of Congo.* Prehosp Disaster Med, 2011. **26**(6): p. 408-13.
15. Human Security Report Project, *Human Security Report 2012: Sexual Violence, Education, and War: Beyond the Mainstream Narrative*. 2012, Vancouver: Human Security Press.
16. Bartels, S., et al., *Militarized Sexual Violence in South Kivu, Democratic Republic of Congo.* J Interpers Violence, 2012.
17. World Health Organization (WHO), *Executive Summary: A research agenda for sexual violence in humanitarian, conflict and post-conflict settings*. 2012, World Health Organization.
18. Koffi, T., *Cote d'Ivoire - l'agonie du jardin du grand reve au desastre*. 2006, Abidjan: CEDA/NEI.
19. Hellweg, J., *Hunting the Ethical State: The Benkadi Movement of Cote d'Ivoire*. 2011: University of Chicago Press.
20. McGovern, M., *Making War in Cote d'Ivoire*. 2011: C Hurst & Co Publishers Ltd.
21. Balint-Kurti, D., *Cote d'Ivoire's Force Nouvelles*, in *Chatham House: Africa Programme Armed Non-State Actors Series*. 2007, Chatham House: London.

For peer review only - http://bmjopen.bmj.com/site/about/guidelines.xhtml

22. Human Rights Watch (HRW), *"A Long Way from Reconciliation" - Abusive Military Crackdown in Response to Security Threats in Cote d'Ivoire*. 2012, Human Rights Watch: New York.
23. United Nations Development Programme (UNDP), *The conflict in Cote d'Ivoire and its effect on West African countries: A perspective from the ground*. 2011, UNDP, Regional Bureau for Africa.
24. IRIN Africa, *Analysis: The dynamics of inter-communal violence in Mali*, in *IRIN - Humanitarian news and analysis*. 2013, UN Office for the Coordination of Humanitarian Affairs.
25. Garcia-Moreno, C., et al., *Prevalence of intimate partner violence: findings from the WHO multi-country study on women's health and domestic violence.* Lancet, 2006. **368**(9543): p. 1260-9.
26. Zimmerman, C., et al., *Asylum-Seeking Women, Violence & Health: Results from a Pilot Study in Scotland and Belgium*. 2009, London School of Hygiene & Tropical Medicine (LSHTM) and Scottish Refugee Council (SRC): London.
27. Abramsky, T., et al., *A community mobilisation intervention to prevent violence against women and reduce HIV/AIDS risk in Kampala, Uganda (the SASA! Study): study protocol for a cluster randomised controlled trial.* Trials, 2012. **13**: p. 96.
28. Mollica, R.F., *Measuring Trauma, Measuring Torture*. 2004, Cambridge: Harvard Program in Refugee Trauma.
29. StataCorp, *Statistical Software: Release 11.1*. 2009, Stata Corporation: College Station, TX (USA).
30. Demographic and Health Survey, *Cote d'Ivoire 2005*, Institut National de la Statistique (INS), Editor. 2005, Measure DHS: Cote d'Ivoire.
31. Garcia-Moreno, C., et al., *WHO Multi-Country Study on Women's Health and Domestic Violence against Women*. 2005, WHO: Genva.
32. Dunkle, K.L., et al., *Perpetration of partner violence and HIV risk behaviour among young men in the rural Eastern Cape, South Africa.* AIDS, 2006. **20**(16): p. 2107-14.
33. Ahonsi, B.A., *Policy Notes: Towards More Informed Responses to Gender Violence and HIV/AIDS in Post-Conflict West African Settings*, N. Afrikainstitutet, Editor. 2010, The Nordic Africa Institute, Conflict, Displacement and Transformation: Uppsala.

For peer review only

For peer review only - http://bmjopen.bmj.com/site/about/guidelines.xhtml

Table 1. Weighted study population demographics and comparison with regional figures.

	Violence survey data		Comparative DHS data from same study regions	
CHARACTERISTICS	WOMEN	MEN	WOMEN	MEN
**Age Range (years)**	*%*		*%*	
15-19	23·7	22·6	24·0	23·3
20-24	20·9	16·8	19·9	20·7
25-29	17·8	15·2	16·4	13·7
30-34	14·0	15·7	12·6	13·0
35-39	10·0	13·4	10·3	11·0
40-44	8·6	9·3	9·9	9·3
45-49	5·0	7·0	6·9	9·1
Total N	*1411*	*1265*	*1407*	*1110*
**Highest educational attainment**				
Primary	19·2	24·0	30·6	31·0
Secondary	11·1	29·9	16·1	35·5
Higher	0·1	1·6	1·0	3·7
Not reported/No schooling	69·6	42·9	52·3	29·8
Total N	*1413*	*1265*	*1407*	*1110*
**Current Living & Partnership Status**				
Living with partner (married/boyfriend/girlfriend)	63·6	51·8	50·3	39·5
Not living with partner (married/boyfriend/girlfriend)	23·6	24·8	15·0	9·1
No partner reported	9·5	21·8	34·7	51·3
Not reported	3·3	1·6	0·0	0·0
Total N	*1413*	*1265*	*1407*	*1110*
**Religion**				
Catholic	13·8	11·2	17·8	18·0
Protestant / Evangelical / Other Christian	33·5	22·.1	32·5	24·9
Muslim	18·2	18·.7	22·.9	21·6
Animist (traditional)	8·6	30·2	25·2	33·8
Other / None reported	18·2	12·9	1·6	1·6
Total N	*1412*	*1265*	*1407*	*1110*
**Number of children**				
None	21·5	45·4	25·0	46·4
1-3	45·2	33·2	39·4	27·5
4-6	25·0	14·7	22·6	15·7
7-9	7·2	4·8	10·1	7·2
10-16	1·2	2·0	2·9	3·1
17+	0	0	0	0·2
Total N	*1413*	*1264*	*1407*	*1110*
**Population in conflict-affected zones**				
Rebel controlled	28·5	30·2	29·.1	31·.3
UN protected	43·9	42·9	36·9	38·7
National army controlled	27·7	26·.9	34·.0	30·0
Total N	*1413*	*1265*	*1407*	*1110*
**Traumatic conflict-related events**				
4 or less experiences	75.5	82.5	n/a	n/a
5 or more experiences	21.5	17.5		
Total N	*1412*	*1263*		

For peer review only

Table 2. Prevalence of interpersonal violence exposures (sexual and physical violence) by sex.

Violence type	WOMEN			MEN			p-value*
**All Violence** (any perpetrator, all respondents)							
Sexual violence	%	(95% CI)	N	%	(95% CI)	N	
Since age 15	32·9	(28·1,38·1)	1408	5·9	(3·6,9·6)	1256	0·00
After the Crisis / Last 12 months	15·1	(11·9,19·0)	1408	0·1	(0·0,0·8)	1256	0·00
Physical violence							
Since age 15	47·6	(41·9,53·4)	1413	38·0	(29·7,47·2)	1265	0·05
After the Crisis / Last 12 months	21·2	(16·0,27·6)	1413	12·2	(8·9,16·4)	1265	0.00
Physical and/or sexual							
Since age 15	57·1	(51·7,62·3)	1413	40·2[1]	(31·0,50·0)	1265	0·01
After the conflict / Last 12 months	29·9	(25·3, 34·9)	1413	12·3[1]	(8·9,16·6)	1265	0·00
**Intimate partner violence** (among ever-partnered)							
Sexual violence							
Lifetime	29·1	(22·3,36·9)	1339	n/d	n/d	n/d	n/d
After the Crisis / Last 12 months	14·9	(11·5,19·2)	1332	n/d	n/d	n/d	n/d
Physical violence (any)							
Lifetime	38·4	(31·7,45·5)	1337	19·8	(12·8,29·4)	1119	0·00
After the Crisis / Last 12 months	20·9	(15·5,27·7)	1339	9·9	(6·8,14·3)	1120	0·00
Physical violence (severe violence)							
Lifetime	23·9	(18·2,30·8)	1337	9·3	(5·9,14·4)	1119	0·00
After the Crisis / Last 12 months	11·6	(6·8, 19·1)	1339	4·2	(2·7,6·5)	1120	0·03
Physical and/or sexual							
Lifetime	49·8	(42·3,57·4)	1339	n/d	n/d	n/d	n/d
After the Crisis / Last 12 months	29·7	(24·9,35·0)	1339	n/d	n/d	n/d	n/d
**Non-partner violence** (among all respondents)							
Sexual violence							
Since age 15	9·9	(7·1,13·8)	1408	5·9	(3·6,9·6)	1256	0·03
After the Crisis / Last 12 months	1·1	(0·6,1·8)	1408	0·1	(0·0,0·8)	1256	0·01
Physical violence							
Since age 15	23·7	(18·4, 29·9)	1412	27·1	(19·9, 35·5)	1257	0·42
After the Crisis / Last 12 months	1·9	(1·3,3·2)	1412	3·6	(2·6, 4·9)	1257	0·02
Physical and/or sexual							
Since age 15	27·7	(22·1,34·0)	1413	29·9	(22·3,38·7)	1265	0·60
After the Crisis / Last 12 months	3·0	(1·8,4·8)	1413	3·6	(2·6,5·0)	1265	0·42
**Child Sexual Abuse** (among all respondents)							
Yes	7·3	(4·9,10·8)	1413	3·3	(1·7,6·5)	1265	0.04
**First sex forced** (among all respondents)							
Yes	14·8	(12·0,18·0)	1333	3·3	(2·4,4·5)	1135	0·00

* p-value denotes difference between women and men
+ Among ever-partnered women and men
n/d = data not available
[1] Does not include sexual violence by an intimate partner.
'All statistics are weighted percentages. Denominators are the sum of the survey weights in the subpopulations of women and men.

For peer review only

Table 3. Prevalence of non-partner violence perpetrator types by conflict time period and sex.

Non-partner violence perpetrators	BEFORE CRISIS (pre-1999)			DURING CRISIS (2000-2007)			AFTER CRISIS (2007-2008)			LIFETIME		
**Sexual violence (>15 years old)** ***Forced or coerced sex***	**Women (%)** (n=1408)	**Men (%)** (n=1256)	p-value	**Women (%)** (n=1408)	**Men (%)** (n=1256)	p-value	**Women (%)** (n=1408)	**Men (%)** (n=1256)	p-value	**Women (%)** (n=1408)	**Men (%)** (n=1256)	p-value
**Any perpetrator (male/female)**	**5·4**	**3·6**	**0·36**	**4·0**	**2·2**	**0·20**	**1·1**	**0·1**	**0·11**	**9·9**	**5·9**	**0·09**
Family member (male)	0·6	0·0	0·25	0·6	0·1	0·26	0·0	0·0	0·52	1·1	0·1	0·11
Family member (female)	0·1	0·2	0·48	0·1	0·2	0·46	0·0	0·0	0·52	0·1	0·5	0·35
Acquaintance (male)	1·2	0·2	0·19	0·8	0·2	0·24	0·1	0·0	0·43	2·0	0·4	0·08
Acquaintance (female)	0·2	2·2	0·02	0·1	1·3	0·06	0·0	0·3	0·08	0·3	3·4	0·00
Acquaintance (sex not reported)	0·1	0·0	0·44	0·1	0·0	0·54	0·0	0·0	0·52	0·2	0·0	0·37
Stranger/Other not identified (male)	2·6	0·0	0·02	1·9	0·1	0·03	0·2	0·0	0·38	4·5	0·1	0·00
Stranger/Other not identified (female)	0·8	1·1	0·58	0·4	0·6	0·58	0·1	0·2	0·57	1·1	1·8	0·41
Stranger/Other not identified (sex unknown)	0·0	0·0	0·52	0·1	0·0	0·45	0·0	0·0	0·52	0·1	0·0	0·45
Combatant / Uniformed Official	0·4	0·7	0·52	0·3	0·2	0·68	0·1	0·0	0·53	0·3	0·2	0·68
**Physical violence (>15 years old)** ***Physically mistreated or hit***	**Women (%)** (n=1412)	**Men (%)** (n=1265)	p-value	**Women (%)** (n=1412)	**Men (%)** (n=1265)	p-value	**Women (%)** (n=1412)	**Men (%)** (n=1265)	p-value	**Women (%)** (n=1412)	**Men (%)** (n=1265)	p-value
**Any perpetrator (male/female)**	**15·4**	**15·2**	**0·36**	**8·9**	**12·6**	**0·13**	**1·9**	**3·6**	**0·02**	**23·6**	**26·9**	**0·27**
Family member (male)	6·4	8·7	0·09	3·2	3·7	0·18	0·9	1·1	0·10	8·9	12·9	0·03
Family member (female)	8·2	2·5	0·00	4·5	1·5	0·00	0·9	1·1	0·16	8·9	3·7	0·01
Acquaintance (male)	1·6	2·4	0·23	0·7	1·4	0·15	0·2	0·8	0·04	2·1	4·4	0·09
Acquaintance (female)	0·9	0·0	0·03	0·4	0·2	0·09	0·1	0·0	0·08	1·1	0·4	0·04
Acquaintance (sex not reported)	0·3	1·7	0·01	0·0	1·2	0·01	0·0	0·6	0·01	0·3	2·9	0·00
Stranger/Other not identified (male)	1·3	2·6	0·01	0·9	1·8	0·04	0·2	0·6	0·18	2·3	4·6	0·01
Stranger/Other not identified (female)	0·2	0·2	0·10	0·0	0·2	0·05	0·1	0·6	0·07	0·2	0·3	0·08
Stranger/Other not identified (sex unknown)	0·1	0·0	0·10	0·0	0·2	0·11	0·1	0·1	0·15	0·1	0·2	0·20
Combatant / Uniformed Official	0·0	0·6	0·04	0·9	4·7	0·00	0·0	0·2	0·61	0·9	5·3	0·00

Family includes: father/mother, father/mother-in-law, and other family members; Acquaintances include: friends, family friends, neighbours, teachers, religious leaders; Strangers/ others include: strangers, individuals not identified; Combatant/uniformed official includes: someone who attacked your village, uniformed official (i.e.-police, gendarme, military), sex unspecified.
* p-value denotes difference between women and men.
ʻ All statistics are weighted percentages. Denominators are the sum of the survey weights in the subpopulations of women and men.

For Peer review only

Figure 1. Prevalence of traumatic experiences by type and timing by sex. (Women: n=1412; Men: n=1263)

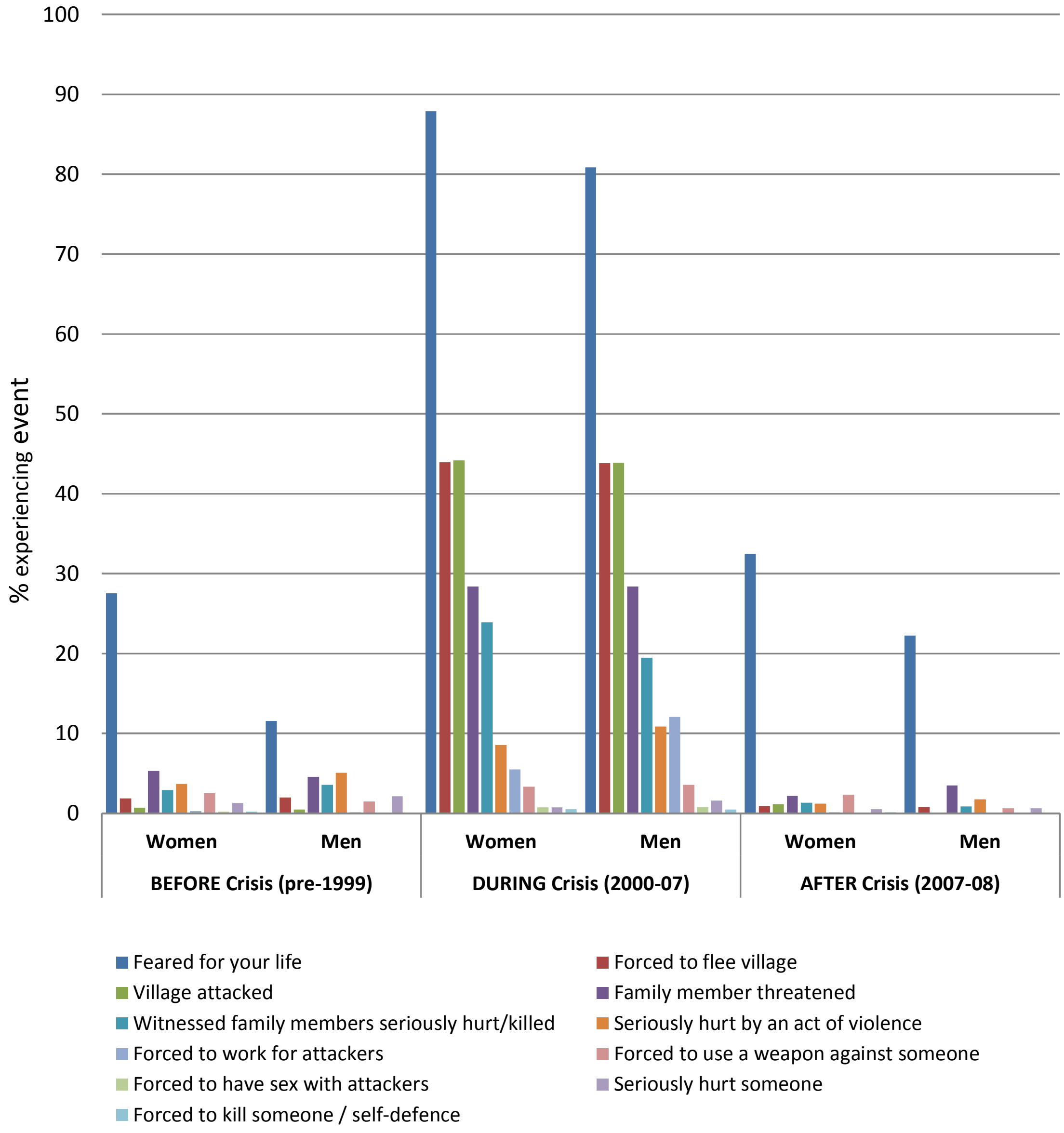

STROBE Statement—checklist of items that should be included in reports of observational studies

	Item No	Recommendation	Page
**Title and abstract**	1	(*a*) Indicate the study's design with a commonly used term in the title or the abstract	**2**
		(*b*) Provide in the abstract an informative and balanced summary of what was done and what was found	**2**
**Introduction**			
Background/rationale	2	Explain the scientific background and rationale for the investigation being reported	**4**
Objectives	3	State specific objectives, including any prespecified hypotheses	**4**
**Methods**			
Study design	4	Present key elements of study design early in the paper	**5-6**
Setting	5	Describe the setting, locations, and relevant dates, including periods of recruitment, exposure, follow-up, and data collection	**5**
Participants	6	(*a*) *Cohort study*—Give the eligibility criteria, and the sources and methods of selection of participants. Describe methods of follow-up<br>*Case-control study*—Give the eligibility criteria, and the sources and methods of case ascertainment and control selection. Give the rationale for the choice of cases and controls<br>*Cross-sectional study*—Give the eligibility criteria, and the sources and methods of selection of participants	**5**
		(*b*) *Cohort study*—For matched studies, give matching criteria and number of exposed and unexposed<br>*Case-control study*—For matched studies, give matching criteria and the number of controls per case	
Variables	7	Clearly define all outcomes, exposures, predictors, potential confounders, and effect modifiers. Give diagnostic criteria, if applicable	**5-6**
Data sources/ measurement	8*	For each variable of interest, give sources of data and details of methods of assessment (measurement). Describe comparability of assessment methods if there is more than one group	**5-6**
Bias	9	Describe any efforts to address potential sources of bias	**6**
Study size	10	Explain how the study size was arrived at	**5**
Quantitative variables	11	Explain how quantitative variables were handled in the analyses. If applicable, describe which groupings were chosen and why	**6**
Statistical methods	12	(*a*) Describe all statistical methods, including those used to control for confounding	**6-7**
		(*b*) Describe any methods used to examine subgroups and interactions	**6**
		(*c*) Explain how missing data were addressed	**6**
		(*d*) *Cohort study*—If applicable, explain how loss to follow-up was addressed<br>*Case-control study*—If applicable, explain how matching of cases and controls was addressed<br>*Cross-sectional study*—If applicable, describe analytical methods taking account of sampling strategy	**5**
		(*e*) Describe any sensitivity analyses	**n/a**

For peer review only

Results			Page
Participants	13*	(a) Report numbers of individuals at each stage of study—eg numbers potentially eligible, examined for eligibility, confirmed eligible, included in the study, completing follow-up, and analysed	**5**
		(b) Give reasons for non-participation at each stage	**5**
		(c) Consider use of a flow diagram	**n/a**
Descriptive data	14*	(a) Give characteristics of study participants (eg demographic, clinical, social) and information on exposures and potential confounders	**8**
		(b) Indicate number of participants with missing data for each variable of interest	**15-17**
		(c) *Cohort study*—Summarise follow-up time (eg, average and total amount)	**n/a**
Outcome data	15*	*Cohort study*—Report numbers of outcome events or summary measures over time	
		Case-control study—Report numbers in each exposure category, or summary measures of exposure	
		Cross-sectional study—Report numbers of outcome events or summary measures	**16-18**
Main results	16	(*a*) Give unadjusted estimates and, if applicable, confounder-adjusted estimates and their precision (eg, 95% confidence interval). Make clear which confounders were adjusted for and why they were included	**n/a**
		(*b*) Report category boundaries when continuous variables were categorized	**n/a**
		(*c*) If relevant, consider translating estimates of relative risk into absolute risk for a meaningful time period	**n/a**
Other analyses	17	Report other analyses done—eg analyses of subgroups and interactions, and sensitivity analyses	**n/a**
**Discussion**			
Key results	18	Summarise key results with reference to study objectives	**3/10**
Limitations	19	Discuss limitations of the study, taking into account sources of potential bias or imprecision. Discuss both direction and magnitude of any potential bias	**10**
Interpretation	20	Give a cautious overall interpretation of results considering objectives, limitations, multiplicity of analyses, results from similar studies, and other relevant evidence	**10-11**
Generalisability	21	Discuss the generalisability (external validity) of the study results	**10-11**
**Other information**			
Funding	22	Give the source of funding and the role of the funders for the present study and, if applicable, for the original study on which the present article is based	**12**

*Give information separately for cases and controls in case-control studies and, if applicable, for exposed and unexposed groups in cohort and cross-sectional studies.

Note: An Explanation and Elaboration article discusses each checklist item and gives methodological background and published examples of transparent reporting. The STROBE checklist is best used in conjunction with this article (freely available on the Web sites of PLoS Medicine at http://www.plosmedicine.org/, Annals of Internal Medicine at http://www.annals.org/, and Epidemiology at http://www.epidem.com/). Information on the STROBE Initiative is available at www.strobe-statement.org.

For peer review only

For peer review only - http://bmjopen.bmj.com/site/about/guidelines.xhtml

Sexual and physical violence in a West-African armed conflict-affected setting: Men's and women's experiences of violence and traumatic events in rural Côte d'Ivoire before, during and after a period of armed conflict

Journal:	*BMJ Open*
Manuscript ID:	bmjopen-2013-003644.R1
Article Type:	Research
Date Submitted by the Author:	05-Nov-2013

Complete List of Authors:	Hossain, Mazeda; London School of Hygiene & Tropical Medicine,<br>Zimmerman, Cathy; London School of Hygiene & Tropical Medicine,<br>Kiss, Ligia; London School of Hygiene & Tropical Medicine,<br>Kone, Drissa; International Rescue Committee,<br>Topolska, Monika; International Rescue Committee,<br>Manan, David; International Rescue Committee,<br>Lehman, Heidi; International Rescue Committee,<br>Watts, Charlotte; London School of Hygiene & Tropical Medicine,

**Primary Subject Heading**:	Global health
Secondary Subject Heading:	Global health, Public health
Keywords:	Violence, Armed conflict, war, Intimate Partner Violence

SCHOLARONE™ Manuscripts

For peer review only

Sexual and physical violence in a West-African armed conflict-affected setting:

Men's and women's experiences of violence and traumatic events in rural Côte d'Ivoire before, during and after a period of armed conflict

Mazeda Hossain, MSc[1]
Cathy Zimmerman, PhD[1]
Ligia Kiss, PhD[1]
Drissa Kone[2]
Monika Topolska[2]
David Manan, MA[2]
Heidi Lehman, MA[2]
Charlotte Watts, PhD[1]

[1] London School of Hygiene & Tropical Medicine
[2] The International Rescue Committee (IRC)

Corresponding author:
Mazeda Hossain
Department of Global Health & Development
London School of Hygiene & Tropical Medicine
15-17 Tavistock Place
London
WC1H 9SH
United Kingdom
Email: Mazeda.Hossain@lshtm.ac.uk

For peer review only

For peer review only - http://bmjopen.bmj.com/site/about/guidelines.xhtml

ABSTRACT

Objective
We assessed women's and men's experiences of gender based violence and other traumatic events in Côte d'Ivoire, a West African conflict-affected setting before, during, and after a period of active armed conflict (2000-2007).

Design
Cross-sectional, household survey.

Setting
12 rural communities directly impacted by the Crisis in Côte d'Ivoire, spanning regions controlled by government forces, rebels, and UN peacekeepers in 2008.

Participants
2,678 women and men aged 15 to 49.

Primary outcome measures
Violence exposures measured since age 15. Questions included intimate partner physical and sexual violence; physical and sexual violence by others (including combatants) and exposure to traumatic events before, during and after the Crisis period (2000-2007).

Results
Physical and/or sexual violence since age 15 was reported by 57.1% women and 40.2% men (p=0.01); 29.9% women and 12.3% men reported past year exposure of any violence. Nearly one in ten women (9.9%) and 5.9% men (p=0.03) were forced to have sex by a non-partner since age 15, and 14.8% women and 3.3% men (p=0.00) reported their first sexual experience was forced. Combatants were rarely reported as sexual violence perpetrators (0.3% women). After the Crisis, intimate partner physical violence was the most frequently reported form of violence and highest among women (20.9% women, 9.9% men, p=0.00). Fearing for your life was reported by women and men before, during and after the Crisis.

Conclusions
Sexual violence in conflict remains a critical international policy concern. However, men and women experience different types of violence before, during and after conflict. In many conflict settings, other forms of violence, including intimate partner violence, may be more widespread than conflict-related sexual violence. Alongside service provision for rape survivors, our findings underscore the need for post-conflict reconstruction efforts to invest in programs to prevent and respond to intimate partner violence and trauma.

For peer review only

For peer review only - http://bmjopen.bmj.com/site/about/guidelines.xhtml

Summary

Article focus

- This study suggests a very different picture from current international policy priorities, which predominantly focuses on conflict-related sexual violence.
- We sought to address the limited population-level data on various types and severity of gender based violence experienced before, during and after an armed conflict period in Côte d'Ivoire, a West African setting among women and men.

Key messages

- More than half of all women (57%) and 40% of men, reported experiencing physical or sexual violence in their lifetime. A third of women (30%) continued to experience intimate partner violence in the 12 months after the Crisis period.
- International attention to conflict-related sexual violence is warranted however, in our study, women experienced the highest levels of violence both within and outside of their homes, and were most likely to report the most severe forms of physical violence by an intimate partner (i.e. dragged, kicked, choked) rather than a combatant. Among women, family members and acquaintances were reported as perpetrators more often than combatants among women.
- Although women reported violence in greater proportions, reconstruction efforts should not ignore men who report high levels of non-partner physical violence. Men are also victims of multiple forms of violence (including sexual violence) and traumas and may be less likely to disclose and receive support.

Strengths and limitations

- This study presents the first temporal violence and trauma prevalence data from regions spanning rebel, government and UN-controlled forces in Côte d'Ivoire.
- This study was not nationally representative however when compared to regional DHS data, the study sample is similar to a regional sample.
- Sexual violence figures should be interpreted with caution as we did not explore the broader range of sexual abuse and response bias is possible.

For peer review only - http://bmjopen.bmj.com/site/about/guidelines.xhtml

INTRODUCTION

The past decade has seen unprecedented recognition of sexual violence in conflict.[1] The UN Security Council alone has issued nine resolutions focused on sexual violence (SV) in conflict and fragile state settings since 2000.[2] Assuming the G8 presidency in 2013, the UK announced its firm commitment to address violence against women and girls and in April launched the *G8 Declaration on Preventing Sexual Violence* with a commitment of £23 million by the G8 nations. In the same year, the UK Foreign Office also announced the *Preventing Sexual Violence Initiative,*[3] towards which the UK Government dedicated £10 million to end sexual violence in conflict.[4] However, there is limited evidence on the prevalence and patterns of violence in conflict-affected settings making it difficult for governments, humanitarian and donor agencies to determine how to target their resources most effectively.[5]

Not surprisingly, robust national level data on the extent of SV are extremely difficult to compile, with current prevalence estimates ranging widely. For example, in the Democratic Republic of Congo (DRC), reports on the extent of conflict-related sexual violence range from 17·8%-39·7% among women and 4%-23.6% among men, due, in part, to methodological differences.[6-8] In the same setting, women also report high levels of violence by an intimate partner (termed intimate partner violence (IPV) or domestic violence), with 35·3% of ever-partnered women reporting sexual partner violence and 56·9% reporting physical partner violence.[9] More recently, data has emerged from Liberia showing high levels of violence and trauma especially among women.[10]

Such data have led to the growing recognition that rape in war is one of numerous forms of violence in conflict-affected settings. Sexual and physical IPV, child sexual abuse, forced marriage, sexual harassment, and rape by non-combatants are also of major concern.[11-14] The most recent Human Security Report (2012) highlighted the discrepancy between the evidence and the international focus on sexual violence in conflict, citing in particular evidence that domestic sexual violence may be more prevalent than rape.[15 12, 16, 17]

Côte d'Ivoire is a West African country that has experienced a protracted conflict, known as *the Crisis*, since a coup d'état in 1999.[18-20] In 2002, a UN-French controlled buffer zone was created, effectively dividing the nation into a rebel controlled north and a government controlled south.[21] The first steps of a peace agreement were brokered in 2007 which was followed by a year of limited conflict-related violence between 2007 and 2008. In 2008 however, the country again entered a period of instability before transitioning to an elected President in 2011.[22] Côte d'Ivoire once considered the 'jewel of West Africa' remains a critical country for regional West African security as it maintains deep ties to neighbouring countries (Mali, Burkina Faso, Ghana, Guinea, Liberia) and other West African nations (Togo, Benin, Sierra Leone, Niger) through migration, trade and remittances. The impact of over a decade of instability and violence is still not known.[23] As the country transitions to the post-conflict period, an understanding of the types of violence and trauma exposures in Côte d'Ivoire may provide insights into programming for health, legal and social sectors both within the country and in neighbouring countries such as Mali who are currently experiencing similar low-level ethnic tensions.[24]

This paper presents the findings from a household survey on women's and men's exposures to interpersonal violence and trauma in 12 rural villages across six administrative districts in Côte d'Ivoire, *prior to* (pre-1999), *during* (2000-2007) and one year *after* a period of active conflict (2008).

For peer review only - http://bmjopen.bmj.com/site/about/guidelines.xhtml

METHODS

Study design and sample

A cross-sectional community survey was conducted in Nov-Dec 2008 among 2,684 respondents (53% women, 47% men) aged 15-49 years in 12 rural communities across six administrative districts in Côte d'Ivoire. These regions included: Yamoussoukro, Daloa, Bouaflé, Bangolo, Danané, and Duekoué. This survey was carried out as a prevalence study prior to the baseline survey of a cluster randomised trial to evaluate the impact of an IPV prevention intervention implemented by a humanitarian organisation in Côte d'Ivoire. The prevalence findings were used to inform the intervention design, which was implemented between 2010-2012 in the same communities.

The study communities were purposively selected based on their accessibility and current relationship with the humanitarian organisation, the International Rescue Committee (IRC). All communities shared similar socio-economic and population size profiles, with residents relying primarily on agriculture as their main income source. The administrative districts spanned regions controlled by the government, rebels, or UN peacekeepers.

Within each community, a representative sample was obtained by first mapping all households to create a sampling list of individuals. All households within each community were eligible to participate. Due to ethical and safety concerns related to disclosure, we did not aim to interview male and female respondents in the same household. Instead, half of the households in the community were randomly allocated to be 'male' respondent households, and the remainder to be 'female' respondent households. In each household, all eligible household members of the same sex who met the eligibility criteria (15-49 years old and resident in the community for at least one year) were invited to participate and be interviewed in private by an interviewer of the same sex. The mapping found that 12,041 individuals lived in the 12 study communities, of which 3,471 were eligible to participate and 2,869 adults completed an interview (83% response rate). Non-response was generally attributed to being out of town, illness or work reasons.

Violence & trauma measures

Behaviourally specific questions were used to ask respondents about their experiences of various acts of violence perpetrated either by an intimate partner (termed 'intimate partner violence', IPV) or by other perpetrators, such as neighbours, relatives, teachers and combatants (termed 'non-partner violence', NPV).

The IPV module drew on survey instruments used internationally to study IPV.[25-27] All ever-partnered participants were asked: "Has your partner ever..." perpetrated a specific act of violence, and if so, when (last 12 months, before the last 12 months) and how often (never, sometimes, often) for each time period. Physical violence acts included being (1) slapped, pushed or (2) hit with something that could hurt you. Severe physical acts were measured by affirmative reports of being (1) kicked, dragged, beaten, (2) choked, burned or (3) threatened with a weapon. Women were also asked about experiences of partner sexual violence, and specifically were asked whether they had been (1) physically forced to have sex; or (2) forced to have sex due to fear.

An individual was considered to have experienced physical partner violence if she/he reported more than one experience of the following acts: hit with a fist or something else, slapped or had something that could hurt thrown at her/him, pushed or shoved. Severe physical violence was defined as reporting at least one experience of the following severe physical violence acts: kicked or dragged, choked or burnt, threatened with a weapon. This categorisation of partner physical violence reflects not only the severity of the act (e.g. the difference between being slapped vs. choked) but also the frequency that the act occurred. It also reflects a more conservative approach that eliminates individuals (women and men) from the overall prevalence who have experienced a

For peer review only - http://bmjopen.bmj.com/site/about/guidelines.xhtml

single act of violence, such as a slap. A woman was considered to have experienced sexual partner violence if she reported one or more experiences of forced sex.

Experiences of non-partner violence measured physical violence and sexual violence as an adult (≥15 years old). For physical violence, questions included: "Since the age of 15, apart from your partner, has anyone ever physically hurt you?" Sexual violence was measured by: "Since the age of 15, apart from your partner, has anyone ever forced you to have sex against your will?" For both types, follow-up questions were asked about the perpetrator, and the timing of the assault in relation to the Crisis (before, during and/or after).

Traumatic events were measured by drawing on the seven domains designated by the Harvard Trauma Questionnaire as common experiences among war-affected populations.[28] Questions were modified to reflect the traumatic experiences potentially associated with conflict-related violence in Côte d'Ivoire. All participants were asked if they had experienced specific events including: feared for your life, village attacked, witnessed family members seriously hurt/killed, forced to work for someone who attacked your village, forced to have sex with someone who attacked your village, forced to flee your village, family member threatened, seriously hurt by an act of violence, forced to use a weapon against someone, and seriously hurt someone. A binary variable was created to capture participants who experiences above median number of events (5 or more).

In each case respondents who reported affirmatively were asked about the timing: in the last 12 months (2007-2008) [After the Crisis], during the Crisis period (1999-2007) [During the Crisis], or before the coup d'état (pre-1999) [Before the Crisis]. To improve recall of event timing, questions were presented with both the years and pivotal historical events such as 'before the coup d'état', 'during the time of Gbabgo' or 'during the Crisis' and 'this year', along with the corresponding years.

Translation, ethics & data collection procedures

The questionnaire was developed in English and French and then translated and back-translated into eight local Ivorian languages. An intensive group translation method was developed by the LSHTM research team where local language speakers translated questions individually and then met as a group (5-10 people) to reach a consensus on the local language interpretation. This interpretation was then checked with the study team and other language groups to ensure that the appropriate and similar meaning was captured across the multiple translations. The final instrument underwent another round of pilot testing and further revision before implementation.

Strict ethical procedures were adopted that recognised possible trauma experienced by the study population and the possibility of renewed violence in the study communities or against field researchers. To ensure the safety of all participants and researchers, all interviewers participated in an intensive three-week training which included ethical and safety training; and all participants were provided access to psychological and medical support.

Face-to-face interviews were conducted in French or local language in a private setting to reduce levels of bias and improve disclosure. Prior to the start of the interviews, consent for the research project was obtained from village leaders, household heads, and individual participants. Quality control measures included the use of multiple checks during the data collection phase and later double-data entry procedures by the data entry team. Ethical clearance for the project was obtained from the LSHTM (London) and the Ministry of Family, Women and Social Affairs (Abidjan) in 2008.

Statistical analysis

The data was double-entered and analysis was completed using Stata 12.[29] Descriptive data analysis was performed using the Stata survey module. Final analysis was conducted among completed

For peer review only - http://bmjopen.bmj.com/site/about/guidelines.xhtml

For peer review only

questionnaires. Prevalence data and 95% confidence intervals were calculated using survey commands to account for clustering at the village level. The design effect due to cluster sampling was assessed using Stata 12 (physical IPV last 12 months ICC=0.04 women; ICC= 0.03 men). Bivariate and sub-group comparisons were calculated using the Wald test where $p<0.05$ was considered statistically significant. Weighted Demographic Health Survey data[30] from the same study regions was examined to compare the representativeness of the study population against a nationally-representative population.

RESULTS

Study population

The majority of study participants were under age 30, with approximately one-quarter of women (23·7%) and men (22·6%) between 15-19 years. (Table 1) Lower literacy levels were reported by women (31%) than men (59%), and less than half of all participants reported basic literacy levels (44%). Most women (87%) said they were in a current relationship with a male partner (i.e. husband, boyfriend), and over half were co-habiting (63·6%). Most men (77%) also reported having a current relationship with either a wife or girlfriend, with half (51.8%) living with their female partner. Almost a third (29%) of partnered women and 12% of partnered men reported their relationship was polygamous, in which the male partner had more than one concurrent wife. (Table 1) Comparisons with the 2005 DHS data suggests that the study population surveyed are representative of the regional population, with similar age breakdowns, levels of educational attainment, number of children and percentage of population living in conflict-affected zones. Differences between partnership status and religion are likely attributable to different definitions between the surveys (Table 1).

<< Table 1 >>

Prevalence of sexual and physical violence exposures

More than half of all women (57·1%) and over a third of all men (40·2%) reported an experience of physical and/or sexual violence since age 15. (Table 2) Approximately one-third of women (29·9%) and 12·3% of men reported physical and/or sexual violence in the 12 month period following the Crisis. The reported levels of physical and/or sexual violence by non-partners were very similar between men and women, with 27·7% of women and 29·9% of men reporting violence by a non-partner since age 15, and 3·0% and 3·6% of women and men respectively reporting abuse in the past year. This highlights that the difference in the overall levels of violence exposure between the sexes may be attributed to the differing levels of IPV experienced by women and men.

<< Table 2 >>

Almost one in ten women (9·9%) reported being forced to have sex by someone other than their intimate partner since age 15, with 1·1% reporting non-partner SV in the past year. For men, the figures were lower but not negligible (5·9% since age 15, 0·1% in the past year). Many women also reported forced sex by a partner, with 29·1% and 14·9% of ever partnered women reporting forced sex ever, and in the past year. (Table 2) In combination, these figures suggest that 32·9% of women have experienced sexual violence since age 15, with most of this sexual violence (24·0% overall) being perpetrated by their intimate partners, and with 5% of women reporting sexual assault by both a partner and other men. Additionally, 14·8% of women and 3·3% of men reported that their first sexual experience was forced (Table 2).

Nearly half of women and over a third of men reported experiencing physical violence since age 15 (47·6% women, 38·0% for men). The levels of physical violence by non-partners were very similar between the sexes over their lifetime (27·7% women, 29·9% men), and in the 12 months following

For peer review only - http://bmjopen.bmj.com/site/about/guidelines.xhtml

the peace agreement (3·0% women, 3·6% men). In contrast, the reported levels of physical violence by a partner were more than twice as high for women compared to men (20·9% versus 9·9%), with women also being more likely to report experiencing severe acts of physical violence by a partner compared to men (23·9% women, 9·3% men, p=0·00) in their lifetime. (Table 2)

Perpetrators

Respondents reported a broad range of physical and sexual violence perpetrators. Table 3 presents the prevalence of non-partner sexual and physical violence perpetrators overall, and broken down according to whether the violence occurred before, during, or after the Crisis periods.

Nearly one in ten women (9·9%) reported sexual violence perpetrated by someone other than their partner, with sexual violence most often perpetrated by male strangers or acquaintances. Only a small percentage of women reported SV perpetrated by a combatant (0·3%). Among men reporting sexual violence from someone other than an intimate partner (5·9%), the most common perpetrators were female acquaintances (3·4% overall), and female strangers (1·8%). (Table 3)

The reported prevalence of non-partner sexual violence was lower after the Crisis period than during or before the Crisis period, potentially as a result of the difference length of time reflected in each measure. In contrast, the prevalence of SV by an intimate partner remained high (14.9% among women after the Crisis). (Table 2)

Men reported higher levels of experiencing physical violence during the Crisis than women (8·9% women, 12·6% men, p=0·02). The perpetrators typically cited were family members for both women and men, except during the Crisis period, when men were more likely than women to report physical assault from combatants (0·9% women, 4·7% men, p=0·00).

<<Table 3>>

Exposure to traumatic conflict-related events

'*Feared for your life'* was the most commonly traumatic event reported with nearly all participants acknowledging having had at least one experience when they feared for their life since age 15 (90% women, 83% men). As expected, levels of all trauma exposures were higher during the active conflict period. Little difference was noted between women and men, except for fearing for one's life, which was higher among women at all time periods. Among all participants, 19.6% reported experiencing five or more traumatic events in their lifetime. (Figure 1)

<< Figure 1>>

For peer review only - http://bmjopen.bmj.com/site/about/guidelines.xhtml

DISCUSSION

As the international community intensifies its focus on violence against women globally, the UK takes up the G8 Presidency with this same priority, and the stability of the West African region is closely monitored, our findings from Côte d'Ivoire encourage increased attention to violence against women and girls and offer important evidence for policies and programming. Our research provides survey-based data on violence against *both* women and men in a conflict setting. Findings from war-affected contexts show that both sexes are subjected to various forms of abuse. However, our data indicates that when resources are limited, a focus on preventing violence against women and girls (whether through direct services for survivors or primary prevention efforts) is reasonable, as women experience violence in significantly greater proportions and are often exposed to more severe abuses. In our study, women experienced the highest levels of violence both within and outside of their homes, and were most likely to report the most severe forms of physical violence by a partner (i.e. dragged, kicked, choked), in addition to experiencing sexual violence by intimate partners and non-partners.

Our findings also confirm that attention to conflict-related sexual violence is warranted. Yet, at the same time, these results emphasise that focussing narrowly on rape in war in all conflict-affected settings is short-sighted. Our data strongly indicate that violence occurs in many forms and is perpetrated by different individuals, in addition to combatants. The most common perpetrators of violence against women in our study appear to be intimate partners, family members and acquaintances, while men report violence from family members, acquaintances, and during the conflict period, from combatants.

While it is clear that focussing limited resources specifically on violence against women is justified, strategies should not, however, exclude violence experienced by men. Importantly, these findings highlight that men are also victims of multiple forms of abuse, including sexual violence. Furthermore, there is reason to believe that men in conflict settings who have experienced violence, especially sexual violence, are likely to have little support and may be less likely to disclose. Men reported higher levels of non-partner physical violence experiences during and after the Crisis. Non-partner sexual violence was higher among women.

This study was limited as it is not nationally representative of Côte d'Ivoire and covers a sub-section of the country. In addition, we only measured forced sex and did not explore the broader range of forms of sexual abuse that may occur. We prioritised comparability between women and men and used survey questions that are more widely used among women.[31] However, we did not pose questions on sexual IPV to men and at the time of implementation there was little research on female-to-male sexual abuse, making data interpretation difficult without a more in-depth understanding of the phenomena. Therefore, the sexual violence prevalence figures should be interpreted with caution, as it is unclear how comparable the data is for women and men. In our study, men identified females that were friends and strangers as perpetrators, however, data was not collected on the relationship or trauma that may have resulted from being forced to have sex. Other research suggests that for men, being forced to have sex by a woman may have different implications than for women and is an area that requires further research.[32] There is also the potential for response bias as given the sensitive nature of the questions participants may be reluctant to report forced sex. Furthermore, although no remuneration was given, there remains the possibility those respondents over-reported, or under-reported, in hopes of receiving services.

Given the range of violence detected in conflict-affected communities, our findings pose a significant challenge to the international community. To truly make inroads in reducing violence against women, programming must address sexual violence against women *in conjunction with* the many

For peer review only - http://bmjopen.bmj.com/site/about/guidelines.xhtml

other types of violence that occur to both women and men. Sexual violence, as our data demonstrates, does not occur in isolation. Indeed, especially in contexts where violence is widespread, such as war-torn areas, forms of violence are likely to be interrelated, potentially exacerbating one another.

Moreover, strategies to address violence occurring in a conflict-affected setting where so many individuals have been exposed to additional traumatic events over the course of their lives, will also need to consider how this range of psychologically damaging circumstances might influence intervention efforts. For example, a majority of study participants reported that they had 'feared for their life', and over 40% of respondents reported being forced to flee their villages due to a violent attack. Promoting recovery and behaviour change (reducing levels of IPV) in a context of fear will undoubtedly be challenging.

Post-conflict reconstruction efforts within West Africa have traditionally focused on security, physical infrastructure and economic development rather than gendered human security issues.[33] However, as Côte d'Ivoire and its neighbour's transition towards peace, the issue of violence against women cannot be ignored. For decision-makers and programmes that have the explicit aim of addressing violence against women in the longer term, this study provides what we hope will be the beginning of a growing evidence-base to foster comprehensive, gender-informed strategies to improve the safety, health and well-being of women, men and children in conflict-affected settings.

For peer review only

Funding
This work was supported by funding from the NoVo Foundation, the Sigrid Rausing Trust and the Economic and Social Research Council (ESRC).

Competing Interests
None declared.

Author Contributions
MH, CZ, CW, LK were responsible for the design and conduct of the study. MH, DK were responsible for field management and study instrument development. MT, HL contributed to national level data collection procedures and oversight. All authors contributed to the development and adaptation of the questionnaires and interpretation of the data. MH was responsible for statistical data analysis. MH, CZ, CW drafted the manuscript which was reviewed and approved by all authors. We are grateful to all of the study respondents who participated in the study. We also gratefully acknowledge the dedicated commitment of IRC country staff and the research field staff.

Ethical approval for this study was received in 2008 from the London School of Hygiene & Tropical Medicine Ethics Committee. Local ethics approval was received from the Ministry of Family, Women and Social Affairs in Côte d'Ivoire.

Data sharing

Preliminary study findings have been presented to the donors, at specialised conferences and several expert group meetings on sexual violence in conflict settings. The data contained in this report is not publically available in a peer-review format.

For peer review only

References

1. Bastick, M., K. Grimm, and R. Kunz, *Sexual Violence in Armed Conflict: Global Overview and Implications for the Security Sector*. 2007, Geneva Centre for the Democratic Control of Armed Forces: Geneva. p. 216.
2. United Nations High Commissioner for Refugees (UNHCR), *Action against Sexual and Gender-Based Violence: An Updated Strategy*. 2011, UNHCR.
3. UK Department for International Development (DfID), *Press release: Helping millions of women and girls access better justice and support against violence*. 2012, DfID: London.
4. UK Foreign & Commonwealth Office and UK Department for International Development. *News Story: UK announces additional funding to address conflict sexual violence*. 2013 [cited; Available from: https://www.gov.uk/government/news/uk-announces-additional-funding-to-address-conflict-sexual-violence.
5. World Health Organization (WHO), *Meeting Report: Sexual Violence in Conflict: Data and Data Collection Methods*. 2008, WHO: Geneva.
6. Casey, S.E., et al., *Care-seeking behavior by survivors of sexual assault in the Democratic Republic of the Congo.* Am J Public Health, 2011. **101**(6): p. 1054-5.
7. Duroch, F., M. McRae, and R.F. Grais, *Description and consequences of sexual violence in Ituri province, Democratic Republic of Congo.* BMC Int Health Hum Rights, 2011. **11**: p. 5.
8. Johnson, K., et al., *Association of sexual violence and human rights violations with physical and mental health in territories of the Eastern Democratic Republic of the Congo.* JAMA, 2010. **304**(5): p. 553-62.
9. Ministère du Plan and Macro International Inc, *Enquête Démographique et de Santé, République Démocratique du Congo 2007*. 2008, Ministère du Plan, Macro International: Calverton, Maryland, USA.
10. Vinck, P. and P.N. Pham, *Association of exposure to intimate-partner physical violence and potentially traumatic war-related events with mental health in Liberia.* Soc Sci Med, 2013. **77**: p. 41-9.
11. Watts, C. and C. Zimmerman, *Violence against women: Global scope and magnitude.* Lancet, 2002. **359**: p. 1232-1237.
12. Geneva Declaration Secretariat, *Global Burden of Armed Violence 2011: Lethal Encounters*. 2011, Cambridge University Press: Cambridge.
13. Peterman, A., T. Palermo, and C. Bredenkamp, *Estimates and determinants of sexual violence against women in the Democratic Republic of Congo.* Am J Public Health, 2011. **101**(6): p. 1060-7.
14. Bartels, S.A., et al., *Sexual violence trends between 2004 and 2008 in South Kivu, Democratic Republic of Congo.* Prehosp Disaster Med, 2011. **26**(6): p. 408-13.
15. Human Security Report Project, *Human Security Report 2012: Sexual Violence, Education, and War: Beyond the Mainstream Narrative*. 2012, Vancouver: Human Security Press.
16. Bartels, S., et al., *Militarized Sexual Violence in South Kivu, Democratic Republic of Congo.* J Interpers Violence, 2012.
17. World Health Organization (WHO), *Executive Summary: A research agenda for sexual violence in humanitarian, conflict and post-conflict settings*. 2012, World Health Organization.
18. Koffi, T., *Côte d'Ivoire - l'agonie du jardin du grand reve au desastre*. 2006, Abidjan: CEDA/NEI.
19. Hellweg, J., *Hunting the Ethical State: The Benkadi Movement of Côte d'Ivoire*. 2011: University of Chicago Press.
20. McGovern, M., *Making War in Côte d'Ivoire*. 2011: C Hurst & Co Publishers Ltd.
21. Balint-Kurti, D., *Côte d'Ivoire's Force Nouvelles*, in *Chatham House: Africa Programme Armed Non-State Actors Series*. 2007, Chatham House: London.

For peer review only - http://bmjopen.bmj.com/site/about/guidelines.xhtml

For peer review only

22. Human Rights Watch (HRW), *"A Long Way from Reconciliation" - Abusive Military Crackdown in Response to Security Threats in Côte d'Ivoire*. 2012, Human Rights Watch: New York.
23. United Nations Development Programme (UNDP), *The conflict in Côte d'Ivoire and its effect on West African countries: A perspective from the ground*. 2011, UNDP, Regional Bureau for Africa.
24. IRIN Africa, *Analysis: The dynamics of inter-communal violence in Mali*, in *IRIN - Humanitarian news and analysis*. 2013, UN Office for the Coordination of Humanitarian Affairs.
25. Garcia-Moreno, C., et al., *Prevalence of intimate partner violence: findings from the WHO multi-country study on women's health and domestic violence.* Lancet, 2006. **368**(9543): p. 1260-9.
26. Zimmerman, C., et al., *Asylum-Seeking Women, Violence & Health: Results from a Pilot Study in Scotland and Belgium*. 2009, London School of Hygiene & Tropical Medicine (LSHTM) and Scottish Refugee Council (SRC): London.
27. Abramsky, T., et al., *A community mobilisation intervention to prevent violence against women and reduce HIV/AIDS risk in Kampala, Uganda (the SASA! Study): study protocol for a cluster randomised controlled trial.* Trials, 2012. **13**: p. 96.
28. Mollica, R.F., *Measuring Trauma, Measuring Torture*. 2004, Cambridge: Harvard Program in Refugee Trauma.
29. StataCorp, *Statistical Software: Release 11.1*. 2009, Stata Corporation: College Station, TX (USA).
30. Demographic and Health Survey, *Côte d'Ivoire 2005*, Institut National de la Statistique (INS), Editor. 2005, Measure DHS: Côte d'Ivoire.
31. Garcia-Moreno, C., et al., *WHO Multi-Country Study on Women's Health and Domestic Violence against Women*. 2005, WHO: Genva.
32. Dunkle, K.L., et al., *Perpetration of partner violence and HIV risk behaviour among young men in the rural Eastern Cape, South Africa.* AIDS, 2006. **20**(16): p. 2107-14.
33. Ahonsi, B.A., *Policy Notes: Towards More Informed Responses to Gender Violence and HIV/AIDS in Post-Conflict West African Settings*, N. Afrikainstitutet, Editor. 2010, The Nordic Africa Institute, Conflict, Displacement and Transformation: Uppsala.

For peer review only

For peer review only - http://bmjopen.bmj.com/site/about/guidelines.xhtml

Table 1. Weighted study population demographics and comparison with regional figures.

	Violence survey data		Comparative DHS data from same study regions	
**CHARACTERISTICS**	**WOMEN**	**MEN**	**WOMEN**	**MEN**
**Age Range (years)**	*%*		*%*	
15-19	23·7	22·6	24·0	23·3
20-24	20·9	16·8	19·9	20·7
25-29	17·8	15·2	16·4	13·7
30-34	14·0	15·7	12·6	13·0
35-39	10·0	13·4	10·3	11·0
40-44	8·6	9·3	9·9	9·3
45-49	5·0	7·0	6·9	9·1
Total N	*1411*	*1265*	*1407*	*1110*
**Highest educational attainment**				
Primary	19·2	24·0	30·6	31·0
Secondary	11·1	29·9	16·1	35·5
Higher	0·1	1·6	1·0	3·7
Not reported/No schooling	69·6	42·9	52·3	29·8
Total N	*1413*	*1265*	*1407*	*1110*
**Current Living & Partnership Status**				
Living with partner (married/boyfriend/girlfriend)	63·6	51·8	50·3	39·5
Not living with partner (married/boyfriend/girlfriend)	23·6	24·8	15·0	9·1
No partner reported	9·5	21·8	34·7	51·3
Not reported	3·3	1·6	0·0	0·0
Total N	*1413*	*1265*	*1407*	*1110*
**Religion**				
Catholic	13·8	11·2	17·8	18·0
Protestant / Evangelical / Other Christian	33·5	22·.1	32·5	24·9
Muslim	18·2	18·.7	22·.9	21·6
Animist (traditional)	8·6	30·2	25·2	33·8
Other / None reported	18·2	12·9	1·6	1·6
Total N	*1412*	*1265*	*1407*	*1110*
**Number of children**				
None	21·5	45·4	25·0	46·4
1-3	45·2	33·2	39·4	27·5
4-6	25·0	14·7	22·6	15·7
7-9	7·2	4·8	10·1	7·2
10-16	1·2	2·0	2·9	3·1
17+	0	0	0	0·2
Total N	*1413*	*1264*	*1407*	*1110*
**Population in conflict-affected zones**				
Rebel controlled	28·5	30·2	29·.1	31·.3
UN protected	43·9	42·9	36·9	38·7
National army controlled	27·7	26.·9	34·.0	30·0
Total N	*1413*	*1265*	*1407*	*1110*
**Traumatic conflict-related events**				
4 or less experiences	75.5	82.5	n/a	n/a
5 or more experiences	21.5	17.5		
Total N	*1412*	*1263*		

For peer review only - http://bmjopen.bmj.com/site/about/guidelines.xhtml

Table 2. Prevalence of interpersonal violence exposures (sexual and physical violence) by sex.

Violence type	WOMEN			MEN			p-value*
**All Violence** (any perpetrator, all respondents)							
Sexual violence	%	(95% CI)	N	%	(95% CI)	N	
Since age 15	32·9	(28·1,38·1)	1408	5·9	(3·6,9·6)	1256	0·00
After the Crisis / Last 12 months	15·1	(11·9,19·0)	1408	0·1	(0·0,0·8)	1256	0·00
Physical violence							
Since age 15	47·6	(41·9,53·4)	1413	38·0	(29·7,47·2)	1265	0·05
After the Crisis / Last 12 months	21·2	(16·0,27·6)	1413	12·2	(8·9,16·4)	1265	0.00
Physical and/or sexual							
Since age 15	57·1	(51·7,62·3)	1413	40·2[1]	(31·0,50·0)	1265	0·01
After the conflict / Last 12 months	29·9	(25·3, 34·9)	1413	12·3[1]	(8·9,16·6)	1265	0·00
**Intimate partner violence** (among ever-partnered)							
Sexual violence							
Lifetime	29·1	(22·3,36·9)	1339	n/d	n/d	n/d	n/d
After the Crisis / Last 12 months	14·9	(11·5,19·2)	1332	n/d	n/d	n/d	n/d
Physical violence (any)							
Lifetime	38·4	(31·7,45·5)	1337	19·8	(12·8,29·4)	1119	0·00
After the Crisis / Last 12 months	20·9	(15·5,27·7)	1339	9·9	(6·8,14·3)	1120	0·00
Physical violence (severe violence)							
Lifetime	23·9	(18·2,30·8)	1337	9·3	(5·9,14·4)	1119	0·00
After the Crisis / Last 12 months	11·6	(6·8, 19·1)	1339	4·2	(2·7,6·5)	1120	0·03
Physical and/or sexual							
Lifetime	49·8	(42·3,57·4)	1339	n/d	n/d	n/d	n/d
After the Crisis / Last 12 months	29·7	(24·9,35·0)	1339	n/d	n/d	n/d	n/d
**Non-partner violence** (among all respondents)							
Sexual violence							
Since age 15	9·9	(7·1,13·8)	1408	5·9	(3·6,9·6)	1256	0·03
After the Crisis / Last 12 months	1·1	(0·6,1·8)	1408	0·1	(0·0,0·8)	1256	0·01
Physical violence							
Since age 15	23·7	(18·4, 29·9)	1412	27·1	(19·9, 35·5)	1257	0·42
After the Crisis / Last 12 months	1·9	(1·3,3·2)	1412	3·6	(2·6, 4·9)	1257	0·02
Physical and/or sexual							
Since age 15	27·7	(22·1,34·0)	1413	29·9	(22·3,38·7)	1265	0·60
After the Crisis / Last 12 months	3·0	(1·8,4·8)	1413	3·6	(2·6,5·0)	1265	0·42
**Child Sexual Abuse** (among all respondents)							
Yes	7·3	(4·9,10·8)	1413	3·3	(1·7,6·5)	1265	0.04
**First sex forced** (among all respondents)							
Yes	14·8	(12·0,18·0)	1333	3·3	(2·4,4·5)	1135	0·00

* p-value denotes difference between women and men
+ Among ever-partnered women and men
n/d = data not available
[1] Does not include sexual violence by an intimate partner.
'All statistics are weighted percentages. Denominators are the sum of the survey weights in the subpopulations of women and men.

For peer review only

For peer review only - http://bmjopen.bmj.com/site/about/guidelines.xhtml

Table 3. Prevalence of non-partner violence perpetrator types by conflict time period and sex.

Non-partner violence perpetrators	BEFORE CRISIS (pre-1999)			DURING CRISIS (2000-2007)			AFTER CRISIS (2007-2008)			LIFETIME		
**Sexual violence (>15 years old)** ***Forced or coerced sex***	**Women (%)** (n=1408)	**Men (%)** (n=1256)	p-value	**Women (%)** (n=1408)	**Men (%)** (n=1256)	p-value	**Women (%)** (n=1408)	**Men (%)** (n=1256)	p-value	**Women (%)** (n=1408)	**Men (%)** (n=1256)	p-value
**Any perpetrator (male/female)**	**5·4**	**3·6**	**0·36**	**4·0**	**2·2**	**0·20**	**1·1**	**0·1**	**0·11**	**9·9**	**5·9**	**0·09**
Family member (male)	0·6	0·0	0·25	0·6	0·1	0·26	0·0	0·0	0·52	1·1	0·1	0·11
Family member (female)	0·1	0·2	0·48	0·1	0·2	0·46	0·0	0·0	0·52	0·1	0·5	0·35
Acquaintance (male)	1·2	0·2	0·19	0·8	0·2	0·24	0·1	0·0	0·43	2·0	0·4	0·08
Acquaintance (female)	0·2	2·2	0·02	0·1	1·3	0·06	0·0	0·3	0·08	0·3	3·4	0·00
Acquaintance (sex not reported)	0·1	0·0	0·44	0·1	0·0	0·54	0·0	0·0	0·52	0·2	0·0	0·37
Stranger/Other not identified (male)	2·6	0·0	0·02	1·9	0·1	0·03	0·2	0·0	0·38	4·5	0·1	0·00
Stranger/Other not identified (female)	0·8	1·1	0·58	0·4	0·6	0·58	0·1	0·2	0·57	1·1	1·8	0·41
Stranger/Other not identified (sex unknown)	0·0	0·0	0·52	0·1	0·0	0·45	0·0	0·0	0·52	0·1	0·0	0·45
Combatant / Uniformed Official	0·4	0·7	0·52	0·3	0·2	0·68	0·1	0·0	0·53	0·3	0·2	0·68
**Physical violence (>15 years old)** ***Physically mistreated or hit***	**Women (%)** (n=1412)	**Men (%)** (n=1265)	p-value	**Women (%)** (n=1412)	**Men (%)** (n=1265)	p-value	**Women (%)** (n=1412)	**Men (%)** (n=1265)	p-value	**Women (%)** (n=1412)	**Men (%)** (n=1265)	p-value
**Any perpetrator (male/female)**	**15·4**	**15·2**	**0·36**	**8·9**	**12·6**	**0·13**	**1·9**	**3·6**	**0·02**	**23·6**	**26·9**	**0·27**
Family member (male)	6·4	8·7	0·09	3·2	3·7	0·18	0·9	1·1	0·10	8·9	12·9	0·03
Family member (female)	8·2	2·5	0·00	4·5	1·5	0·00	0·9	1·1	0·16	8·9	3·7	0·01
Acquaintance (male)	1·6	2·4	0·23	0·7	1·4	0·15	0·2	0·8	0·04	2·1	4·4	0·09
Acquaintance (female)	0·9	0·0	0·03	0·4	0·2	0·09	0·1	0·0	0·08	1·1	0·4	0·04
Acquaintance (sex not reported)	0·3	1·7	0·01	0·0	1·2	0·01	0·0	0·6	0·01	0·3	2·9	0·00
Stranger/Other not identified (male)	1·3	2·6	0·01	0·9	1·8	0·04	0·2	0·6	0·18	2·3	4·6	0·01
Stranger/Other not identified (female)	0·2	0·2	0·10	0·0	0·2	0·05	0·1	0·6	0·07	0·2	0·3	0·08
Stranger/Other not identified (sex unknown)	0·1	0·0	0·10	0·0	0·2	0·11	0·1	0·1	0·15	0·1	0·2	0·20
Combatant / Uniformed Official	0·0	0·6	0·04	0·9	4·7	0·00	0·0	0·2	0·61	0·9	5·3	0·00

Family includes: father/mother, father/mother-in-law, and other family members; Acquaintances include: friends, family friends, neighbours, teachers, religious leaders; Strangers/ others include: strangers, individuals not identified; Combatant/uniformed official includes: someone who attacked your village, uniformed official (i.e.-police, gendarme, military), sex unspecified.

* p-value denotes difference between women and men.

ʻ All statistics are weighted percentages. Denominators are the sum of the survey weights in the subpopulations of women and men.

For Peer review only

For peer review only - http://bmjopen.bmj.com/site/about/guidelines.xhtml

Figure legend

Figure 1. Prevalence of traumatic experiences by type of event, timing in relation to the Crisis and by sex.
(Women: n=1412; Men: n=1263)

For peer review only

For peer review only - http://bmjopen.bmj.com/site/about/guidelines.xhtml

STROBE Statement—checklist of items that should be included in reports of observational studies

	Item No	Recommendation	Page
**Title and abstract**	1	(*a*) Indicate the study's design with a commonly used term in the title or the abstract	**2**
		(*b*) Provide in the abstract an informative and balanced summary of what was done and what was found	**2**
**Introduction**			
Background/rationale	2	Explain the scientific background and rationale for the investigation being reported	**4**
Objectives	3	State specific objectives, including any prespecified hypotheses	**4**
**Methods**			
Study design	4	Present key elements of study design early in the paper	**5-6**
Setting	5	Describe the setting, locations, and relevant dates, including periods of recruitment, exposure, follow-up, and data collection	**5**
Participants	6	(*a*) *Cohort study*—Give the eligibility criteria, and the sources and methods of selection of participants. Describe methods of follow-up<br>*Case-control study*—Give the eligibility criteria, and the sources and methods of case ascertainment and control selection. Give the rationale for the choice of cases and controls<br>*Cross-sectional study*—Give the eligibility criteria, and the sources and methods of selection of participants	**5**
		(*b*) *Cohort study*—For matched studies, give matching criteria and number of exposed and unexposed<br>*Case-control study*—For matched studies, give matching criteria and the number of controls per case	
Variables	7	Clearly define all outcomes, exposures, predictors, potential confounders, and effect modifiers. Give diagnostic criteria, if applicable	**5-6**
Data sources/ measurement	8*	For each variable of interest, give sources of data and details of methods of assessment (measurement). Describe comparability of assessment methods if there is more than one group	**5-6**
Bias	9	Describe any efforts to address potential sources of bias	**6**
Study size	10	Explain how the study size was arrived at	**5**
Quantitative variables	11	Explain how quantitative variables were handled in the analyses. If applicable, describe which groupings were chosen and why	**6**
Statistical methods	12	(*a*) Describe all statistical methods, including those used to control for confounding	**6-7**
		(*b*) Describe any methods used to examine subgroups and interactions	**6**
		(*c*) Explain how missing data were addressed	**6**
		(*d*) *Cohort study*—If applicable, explain how loss to follow-up was addressed<br>*Case-control study*—If applicable, explain how matching of cases and controls was addressed<br>*Cross-sectional study*—If applicable, describe analytical methods taking account of sampling strategy	**5**
		(*e*) Describe any sensitivity analyses	**n/a**

For peer review only

Results			Page
Participants	13*	(a) Report numbers of individuals at each stage of study—eg numbers potentially eligible, examined for eligibility, confirmed eligible, included in the study, completing follow-up, and analysed	**5**
		(b) Give reasons for non-participation at each stage	**5**
		(c) Consider use of a flow diagram	**n/a**
Descriptive data	14*	(a) Give characteristics of study participants (eg demographic, clinical, social) and information on exposures and potential confounders	**8**
		(b) Indicate number of participants with missing data for each variable of interest	**15-17**
		(c) *Cohort study*—Summarise follow-up time (eg, average and total amount)	**n/a**
Outcome data	15*	*Cohort study*—Report numbers of outcome events or summary measures over time	
		Case-control study—Report numbers in each exposure category, or summary measures of exposure	
		Cross-sectional study—Report numbers of outcome events or summary measures	**16-18**
Main results	16	(*a*) Give unadjusted estimates and, if applicable, confounder-adjusted estimates and their precision (eg, 95% confidence interval). Make clear which confounders were adjusted for and why they were included	**n/a**
		(*b*) Report category boundaries when continuous variables were categorized	**n/a**
		(*c*) If relevant, consider translating estimates of relative risk into absolute risk for a meaningful time period	**n/a**
Other analyses	17	Report other analyses done—eg analyses of subgroups and interactions, and sensitivity analyses	**n/a**
**Discussion**			
Key results	18	Summarise key results with reference to study objectives	**3/10**
Limitations	19	Discuss limitations of the study, taking into account sources of potential bias or imprecision. Discuss both direction and magnitude of any potential bias	**10**
Interpretation	20	Give a cautious overall interpretation of results considering objectives, limitations, multiplicity of analyses, results from similar studies, and other relevant evidence	**10-11**
Generalisability	21	Discuss the generalisability (external validity) of the study results	**10-11**
**Other information**			
Funding	22	Give the source of funding and the role of the funders for the present study and, if applicable, for the original study on which the present article is based	**12**

*Give information separately for cases and controls in case-control studies and, if applicable, for exposed and unexposed groups in cohort and cross-sectional studies.

Note: An Explanation and Elaboration article discusses each checklist item and gives methodological background and published examples of transparent reporting. The STROBE checklist is best used in conjunction with this article (freely available on the Web sites of PLoS Medicine at http://www.plosmedicine.org/, Annals of Internal Medicine at http://www.annals.org/, and Epidemiology at http://www.epidem.com/). Information on the STROBE Initiative is available at www.strobe-statement.org.

For peer review only

Sexual and physical violence in a West-African armed conflict-affected setting:

Men's and women's experiences of violence and traumatic events in rural Côte d'Ivoire before, during and after a period of armed conflict

Mazeda Hossain, MSc[1]
Cathy Zimmerman, PhD[1]
Ligia Kiss, PhD[1]
Drissa Kone[2]
Monika Topolska[2]
David Manan, MA[2]
Heidi Lehman, MA[2]
Charlotte Watts, PhD[1]

[1] London School of Hygiene & Tropical Medicine
[2] The International Rescue Committee (IRC)

Corresponding author:
Mazeda Hossain
Department of Global Health & Development
London School of Hygiene & Tropical Medicine
15-17 Tavistock Place
London
WC1H 9SH
United Kingdom
Email: Mazeda.Hossain@lshtm.ac.uk

For peer review only - http://bmjopen.bmj.com/site/about/guidelines.xhtml

ABSTRACT

Objective

~~Sexual violence in conflict is recognized as a crime against humanity yet there is limited population data on its magnitude in different settings and little on the prevalence of other serious forms of violence. Therefore, w~~We ~~measured~~ assessed women's and men's experiences of gender based violence and other traumatic events in Côte d'Ivoire, ~~within~~ a West African conflict-affected setting before, during, and after a period of active armed conflict (2000-2007).

Design

Cross-sectional, household survey.

Setting

12 rural communities directly impacted by the Crisis in Côte d'Ivoire, spanning regions controlled by government forces, rebels, and UN peacekeepers in 2008.

Participants

2,678 women and men aged 15 to 49.

Primary outcome measures

Violence exposures measured since age 15. Questions included intimate partner physical and sexual violence; physical and sexual violence by others (including combatants) and exposure to traumatic events before, during and after the Crisis period (2000-2007).

Results

Physical and/or sexual violence since age 15 was reported by 57.1% women and 40.2% men (p=0.01); 29.9% women and 12.3% men reported past year exposure of any violence. Nearly one in ten women (9.9%) and 5.9% men (p=0.03) were forced to have sex by a non-partner since age 15, and 14.8% women and 3.3% men (p=0.00) reported their first sexual experience was forced. Combatants were rarely reported as sexual violence perpetrators (0.3% women). After the Crisis, intimate partner physical violence was the most frequently reported form of violence and highest among women (20.9% women, 9.9% men, p=0.00). Fearing for your life was reported by women and men before, during and after the Crisis.

Conclusions

Sexual violence in conflict remains a critical international policy concern. However, men and women experience different types of violence before, during and after conflict. In many conflict settings, other forms of violence, including intimate partner violence, may be more widespread than conflict-related sexual violence. Alongside service provision for rape survivors, our findings underscore the need for post-conflict reconstruction efforts to invest in programs to prevent and respond to intimate partner violence and trauma.

For peer review only

For peer review only - http://bmjopen.bmj.com/site/about/guidelines.xhtml

Summary

Article focus

- This study suggests a very different picture from current international policy priorities, which predominantly focuses on conflict-related sexual violence.
- We sought to address the limited population-level data on various types and severity of gender based violence experienced before, during and after an armed conflict period in Côte d'Ivoire, a West African setting among women and men.

Key messages

- More than half of all women (57%) and 40% of men, reported experiencing physical or sexual violence in their lifetime. A third of women (30%) continued to experience intimate partner violence in the 12 months after the Crisis period.
- International attention to conflict-related sexual violence is warranted however, in our study, women experienced the highest levels of violence both within and outside of their homes, and were most likely to report the most severe forms of physical violence by an intimate partner (i.e. dragged, kicked, choked) rather than a combatant. Among women, family members and acquaintances were reported as perpetrators more often than combatants among women.
- Although women reported violence in greater proportions, reconstruction efforts should not ignore men who report high levels of non-partner physical violence. Men are also victims of multiple forms of violence (including sexual violence) and traumas and may be less likely to disclose and receive support.

Strengths and limitations

- This study presents the first temporal violence and trauma prevalence data from regions spanning rebel, government and UN-controlled forces in Côte d'Ivoire.
- This study was not nationally representative however when compared to regional DHS data, the study sample is similar to a regional sample.
- Sexual violence figures should be interpreted with caution as we did not explore the broader range of sexual abuse and response bias is possible.

For peer review only

For peer review only - http://bmjopen.bmj.com/site/about/guidelines.xhtml

INTRODUCTION

The past decade has seen unprecedented recognition of sexual violence in conflict.[1] The UN Security Council alone has issued nine resolutions focused on sexual violence (SV) in conflict and fragile state settings since 2000.[2] Assuming the G8 presidency in 2013, the UK announced its firm commitment to address violence against women and girls and in April launched the *G8 Declaration on Preventing Sexual Violence* with a commitment of £23 million by the G8 nations. In the same year, the UK Foreign Office also announced the *Preventing Sexual Violence Initiative,*[3] towards which the UK Government dedicated £10 million to end sexual violence in conflict.[4] However, there is limited evidence on the prevalence and patterns of violence in conflict-affected settings making it difficult for governments, humanitarian and donor agencies to determine how to target their resources most effectively.[5]

Not surprisingly, robust national level data on the extent of SV are extremely difficult to compile, with current prevalence estimates ranging widely. For example, in the Democratic Republic of Congo (DRC), reports on the extent of conflict-related sexual violence range from 17·8%-39·7% among women and 4%-23.6% among men, due, in part, to methodological differences.[6-8] In the same setting, women also report high levels of violence by an intimate partner (termed intimate partner violence (IPV) or domestic violence), with 35·3% of ever-partnered women reporting sexual partner violence and 56·9% reporting physical partner violence.[9] More recently, data has emerged from Liberia showing high levels of violence and trauma especially among women.[10]

Such data have led to the growing recognition that rape in war is one of numerous forms of violence in conflict-affected settings. Sexual and physical IPV, child sexual abuse, forced marriage, sexual harassment, and rape by non-combatants are also of major concern.[11-14] The most recent Human Security Report (2012) highlighted the discrepancy between the evidence and the international focus on sexual violence in conflict, citing in particular evidence that domestic sexual violence may be more prevalent than rape.[15 12, 16, 17]

Côte d'Ivoire is a West African country that has experienced a protracted conflict, known as *the Crisis*, since a coup d'état in 1999.[18-20] In 2002, a UN-French controlled buffer zone was created, effectively dividing the nation into a rebel controlled north and a government controlled south.[21] The first steps of a peace agreement were brokered in 2007 which was followed by a year of limited conflict-related violence between 2007 and 2008. In 2008 however, the country again entered a period of instability before transitioning to an elected President in 2011.[22] Côte d'Ivoire once considered the 'jewel of West Africa' remains a critical country for regional West African security as it maintains deep ties to neighbouring countries (Mali, Burkina Faso, Ghana, Guinea, Liberia) and other West African nations (Togo, Benin, Sierra Leone, Niger) through migration, trade and remittances. The impact of over a decade of instability and violence is still not known.[23] As the country transitions to the post-conflict period, an understanding of the types of violence and trauma exposures in Côte d'Ivoire may provide insights into programming for health, legal and social sectors both within the country and in neighbouring countries such as Mali who are currently experiencing similar low-level ethnic tensions.[24]

This paper presents the findings from a household survey on women's and men's exposures to interpersonal violence and trauma in 12 rural villages across six administrative districts in Côte d'Ivoire, *prior to* (pre-1999), *during* (2000-2007) and one year *after* a period of active conflict (2008).

For peer review only - http://bmjopen.bmj.com/site/about/guidelines.xhtml

METHODS

Study design and sample

A cross-sectional community survey was conducted in Nov-Dec 2008 among 2,684 respondents (53% women, 47% men) aged 15-49 years in 12 rural communities across six administrative districts in Côte d'Ivoire. These regions included: Yamoussoukro, Daloa, Bouaflé, Bangolo, Danané, and Duekoué. This survey was carried out as a prevalence study prior to the baseline survey of ~~part of a formative assessment for~~ a cluster randomised trial to evaluate the impact of an IPV prevention intervention implemented by a humanitarian organisation in Côte d'Ivoire. The prevalence findings were used to inform the intervention design, which was implemented between 2010-2012 in the same communities.

The study communities were purposively selected based on their accessibility and current relationship with the humanitarian organisation, the International Rescue Committee (IRC). All communities shared similar socio-economic and population size profiles, with residents relying primarily on agriculture as their main income source. The administrative districts spanned regions controlled by the government, rebels, or UN peacekeepers.

Within each community, a representative sample was obtained by first mapping all households to create a sampling list of individuals. All households within each community were eligible to participate. Due to ethical and safety concerns related to disclosure, we did not aim to interview male and female respondents in the same household. Instead, half of the households in the community were randomly allocated to be 'male' respondent households, and the remainder to be 'female' respondent households. In each household, all eligible household members of the same sex who met the eligibility criteria (15-49 years old and resident in the community for at least one year) were invited to participate and be interviewed in private by an interviewer of the same sex. The mapping found that 12,041 individuals lived in the 12 study communities, of which 3,471 were eligible to participate and 2,869 adults completed an interview (83% response rate). Non-response was generally attributed to being out of town, illness or work reasons.

Violence & trauma measures

Behaviourally specific questions were used to ask respondents about their experiences of various acts of violence perpetrated either by an intimate partner (termed 'intimate partner violence', IPV) or by other perpetrators, such as neighbours, relatives, teachers and combatants (termed 'non-partner violence', NPV).

The IPV module drew on survey instruments used internationally to study IPV.[25-27] All ever-partnered participants were asked: "Has your partner ever..." perpetrated a specific act of violence, and if so, when (last 12 months, before the last 12 months) and how often (never, sometimes, often) for each time period. Physical violence acts included being (1) slapped, pushed or (2) hit with something that could hurt you. Severe physical acts were measured by affirmative reports of being (1) kicked, dragged, beaten, (2) choked, burned or (3) threatened with a weapon. Women were also asked about experiences of partner sexual violence, and specifically were asked whether they had been (1) physically forced to have sex; or (2) forced to have sex due to fear.

An individual was considered to have experienced physical partner violence if she/he reported more than one experience of the following acts: hit with a fist or something else, slapped or had something that could hurt thrown at her/him, pushed or shoved. Severe physical violence was defined as reporting at least one experience of the following severe physical violence acts: kicked or dragged, choked or burnt, threatened with a weapon. This categorisation of partner physical violence reflects not only the severity of the act (e.g. the difference between being slapped vs. choked) but also the frequency that the act occurred. It also reflects a more conservative approach

For peer review only - http://bmjopen.bmj.com/site/about/guidelines.xhtml

that eliminates individuals (women and men) from the overall prevalence who have experienced a single act of violence, such as a slap. A woman was considered to have experienced sexual partner violence if she reported one or more experiences of forced sex.

Experiences of non-partner violence measured physical violence and sexual violence as an adult (≥15 years old). For physical violence, questions included: "Since the age of 15, apart from your partner, has anyone ever physically hurt you?" Sexual violence was measured by: "Since the age of 15, apart from your partner, has anyone ever forced you to have sex against your will?" For both types, follow-up questions were asked about the perpetrator, and the timing of the assault in relation to the Crisis (before, during and/or after).

Traumatic events were measured by drawing on the seven domains designated by the Harvard Trauma Questionnaire as common experiences among war-affected populations.[28] Questions were modified to reflect the traumatic experiences potentially associated with conflict-related violence in Côte d'Ivoire. All participants were asked if they had experienced specific events including: feared for your life, village attacked, witnessed family members seriously hurt/killed, forced to work for someone who attacked your village, forced to have sex with someone who attacked your village, forced to flee your village, family member threatened, seriously hurt by an act of violence, forced to use a weapon against someone, and seriously hurt someone. A binary variable was created to capture participants who experiences above median number of events (5 or more).

In each case respondents who reported affirmatively were asked about the timing: in the last 12 months (2007-2008) [After the Crisis], during the Crisis period (1999-2007) [During the Crisis], or before the coup d'état (pre-1999) [Before the Crisis]. To improve recall of event timing, questions were presented with both the years and pivotal historical events such as 'before the coup d'état', 'during the time of Gbabgo' or 'during the Crisis' and 'this year', along with the corresponding years.

Translation, ethics & data collection procedures

The questionnaire was developed in English and French and then translated and back-translated into eight local Ivorian languages. An intensive group translation method was developed by the LSHTM research team where local language speakers translated questions individually and then met as a group (5-10 people) to reach a consensus on the local language interpretation. This interpretation was then checked with the study team and other language groups to ensure that the appropriate and similar meaning was captured across the multiple translations. The final instrument underwent another round of pilot testing and further revision before implementation.

Strict ethical procedures were adopted that recognised possible trauma experienced by the study population and the possibility of renewed violence in the study communities or against field researchers. To ensure the safety of all participants and researchers, all interviewers participated in an intensive three-week training which included ethical and safety training; and all participants were provided access to psychological and medical support.

Face-to-face interviews were conducted in French or local language in a private setting to reduce levels of bias and improve disclosure. Prior to the start of the interviews, consent for the research project was obtained from village leaders, household heads, and individual participants. Quality control measures included the use of multiple checks during the data collection phase and later double-data entry procedures by the data entry team. Ethical clearance for the project was obtained from the LSHTM (London) and the Ministry of Family, Women and Social Affairs (Abidjan) in 2008.

Statistical analysis

For peer review only - http://bmjopen.bmj.com/site/about/guidelines.xhtml

The data was double-entered and analysis was completed using Stata 12.[29] Descriptive data analysis was performed using the Stata survey module. Final analysis was conducted among completed questionnaires. Prevalence data and 95% confidence intervals were calculated using survey commands to account for clustering at the village level. The design effect due to cluster sampling was assessed using Stata 12 (physical IPV last 12 months ICC=0.04 women; ICC= 0.03 men). Bivariate and sub-group comparisons were calculated using the Wald test where $p<0.05$ was considered statistically significant. Weighted Demographic Health Survey data[30] from the same study regions was examined to compare the representativeness of the study population against a nationally-representative population.

For peer review only

RESULTS

Study population

The majority of study participants were under age 30, with approximately one-quarter of women (23·7%) and men (22·6%) between 15-19 years. (Table 1) Lower literacy levels were reported by women (31%) than men (59%), and less than half of all participants reported basic literacy levels (44%). Most women (87%) said they were in a current relationship with a male partner (i.e. husband, boyfriend), and over half were co-habiting (63·6%). Most men (77%) also reported having a current relationship with either a wife or girlfriend, with half (51.8%) living with their female partner. Almost a third (29%) of partnered women and 12% of partnered men reported their relationship was polygamous, in which the male partner had more than one concurrent wife. (Table 1) Comparisons with the 2005 DHS data suggests that the study population surveyed are representative of the regional population, with similar age breakdowns, levels of educational attainment, number of children and percentage of population living in conflict-affected zones. Differences between partnership status and religion are likely attributable to different definitions between the surveys (Table 1).

<< Table 1 >>

Prevalence of sexual and physical violence exposures

More than half of all women (57·1%) and over a third of all men (40·2%) reported an experience of physical and/or sexual violence since age 15. (Table 2) Approximately one-third of women (29·9%) and 12·3% of men reported physical and/or sexual violence in the 12 month period following the Crisis. The reported levels of physical and/or sexual violence by non-partners were very similar between men and women, with 27·7% of women and 29·9% of men reporting violence by a non-partner since age 15, and 3·0% and 3·6% of women and men respectively reporting abuse in the past year. This highlights that the difference in the overall levels of violence exposure between the sexes may be attributed to the differing levels of IPV experienced by women and men.

<< Table 2 >>

Almost one in ten women (9·9%) reported being forced to have sex by someone other than their intimate partner since age 15, with 1·1% reporting non-partner SV in the past year. For men, the figures were lower but not negligible (5·9% since age 15, 0·1% in the past year). Many women also reported forced sex by a partner, with 29·1% and 14·9% of ever partnered women reporting forced sex ever, and in the past year. (Table 2) In combination, these figures suggest that 32·9% of women have experienced sexual violence since age 15, with most of this sexual violence (24·0% overall) being perpetrated by their intimate partners, and with 5% of women reporting sexual assault by both a partner and other men. Additionally, 14·8% of women and 3·3% of men reported that their first sexual experience was forced (Table 2).

Nearly half of women and over a third of men reported experiencing physical violence since age 15 (47·6% women, 38·0% for men). The levels of physical violence by non-partners were very similar between the sexes over their lifetime (27·7% women, 29·9% men), and in the 12 months following the peace agreement (3·0% women, 3·6% men). In contrast, the reported levels of physical violence by a partner were more than twice as high for women compared to men (20·9% versus 9·9%), with women also being more likely to report experiencing severe acts of physical violence by a partner compared to men (23·9% women, 9·3% men, p=0·00) in their lifetime. (Table 2)

Perpetrators

For peer review only - http://bmjopen.bmj.com/site/about/guidelines.xhtml

Respondents reported a broad range of physical and sexual violence perpetrators. Table 3 presents the prevalence of non-partner sexual and physical violence perpetrators overall, and broken down according to whether the violence occurred before, during, or after the Crisis periods.

Nearly one in ten women (9·9%) reported sexual violence perpetrated by someone other than their partner, with sexual violence most often perpetrated by male strangers or acquaintances. Only a small percentage of women reported SV perpetrated by a combatant (0·3%). Among men reporting sexual violence from someone other than an intimate partner ~~Fewer men~~ (5·9%), ~~reported sexual violence from someone other than their partner, with~~ the most common perpetrators were ~~being~~ female acquaintances (3·4% overall), and female strangers (1·8%). (Table 3)

The reported prevalence of non-partner sexual violence was lower after the Crisis period than during or before the Crisis period, potentially as a result of the difference length of time reflected in each measure. In contrast, the prevalence of SV by an intimate partner remained high (14.9% among women after the Crisis). (Table 2)

Men reported higher levels of experiencing physical violence during the Crisis than women (8·9% women, 12·6% men, p=0·02). The perpetrators typically cited were family members for both women and men, except during the Crisis period, when men were more likely than women to report physical assault from combatants (0·9% women, 4·7% men, p=0·00).

<<Table 3>>

Exposure to traumatic conflict-related events

'*Feared for your life'* was the most commonly traumatic event reported with nearly all participants acknowledging having had at least one experience when they feared for their life since age 15 (90% women, 83% men). As expected, levels of all trauma exposures were higher during the active conflict period. Little difference was noted between women and men, except for fearing for one's life, which was higher among women at all time periods. Among all participants, 19.6% reported experiencing five or more traumatic events in their lifetime. (Figure 1)

<< Figure 1>>

For peer review only

For peer review only - http://bmjopen.bmj.com/site/about/guidelines.xhtml

DISCUSSION

As the international community intensifies its focus on violence against women globally, the UK takes up the G8 Presidency with this same priority, and the stability of the West African region is closely monitored, our findings from Côte d'Ivoire encourage increased attention to violence against women and girls and offer important evidence for policies and programming. Our research provides survey-based data on violence against *both* women and men in a conflict setting. Findings from war-affected contexts show that both sexes are subjected to various forms of abuse. However, our data indicates that when resources are limited, a focus on preventing violence against women and girls (whether through direct services for survivors or primary prevention efforts) is reasonable ~~at times~~, as women experience violence in significantly greater proportions and are often exposed to more severe abuses. In our study, women experienced the highest levels of violence both within and outside of their homes, and were most likely to report the most severe forms of physical violence by a partner (i.e. dragged, kicked, choked), in addition to experiencing sexual violence by intimate partners and non-partners.

Our findings also confirm that attention to conflict-related sexual violence is warranted. Yet, at the same time, these results emphasise that focussing narrowly on rape in war in all conflict-affected settings is short-sighted. Our data strongly indicate that violence occurs in many forms and is perpetrated by different individuals, in addition to combatants. The most common perpetrators of violence against women in our study appear to be intimate partners, family members and acquaintances, while men report violence from family members, acquaintances, and during the conflict period, from combatants.

While it is clear that focussing limited resources specifically on violence against women is justified, strategies should not, however, exclude violence experienced by men. Importantly, these findings highlight that men are also victims of multiple forms of abuse, including sexual violence. Furthermore, there is reason to believe that men in conflict settings who have experienced violence, especially sexual violence, are likely to have little support and may be less likely to disclose. Men reported higher levels of non-partner physical violence experiences during and after the Crisis. Non-partner sexual violence was higher among women.

This study was limited as it is not nationally representative of Côte d'Ivoire and covers a sub-section of the country. In addition, we only measured forced sex~~,~~ and did not explore the broader range of forms of sexual abuse that may occur. We prioritised comparability between women and men and used survey questions that are more widely used among women.[31] However, we did not pose questions on sexual IPV to men and at the time of implementation there was little research on female-to-male sexual abuse, making data interpretation difficult without a more in-depth understanding of the phenomena. Therefore, the sexual violence prevalence figures should be interpreted with caution, as it is unclear how comparable the data is for women and men. In our study, men identified females that were friends and strangers as perpetrators, however, data was not collected on the relationship or trauma that may have resulted from being forced to have sex. Other research suggests that for men, being forced to have sex by a woman may have different implications than for women and is an area that requires further research.[32] There is also the potential for response bias as given the sensitive nature of the questions participants may be reluctant to report forced sex. Furthermore, although no remuneration was given, there remains the possibility those respondents over-reported, or under-reported, in hopes of receiving services.

Given the range of violence detected in conflict-affected communities, our findings pose a significant challenge to the international community. To truly make inroads in reducing violence against women, programming must address sexual violence against women *in conjunction with* the many

For peer review only - http://bmjopen.bmj.com/site/about/guidelines.xhtml

other types of violence that occur to both women and men. Sexual violence, as our data demonstrates, does not occur in isolation. Indeed, especially in contexts where violence is widespread, such as war-torn areas, forms of violence are likely to be interrelated, potentially exacerbating one another.

Moreover, strategies to address violence occurring in a conflict-affected setting where so many individuals have been exposed to additional traumatic events over the course of their lives, will also need to consider how this range of psychologically damaging circumstances might influence intervention efforts. For example, a majority of study participants reported that they had 'feared for their life', and over 40% of respondents reported being forced to flee their villages due to a violent attack. Promoting recovery and behaviour change (reducing levels of IPV) in a context of fear will undoubtedly be challenging.

Post-conflict reconstruction efforts within West Africa have traditionally focused on security, physical infrastructure and economic development rather than gendered human security issues.[33] However, as Côte d'Ivoire and its neighbour's transition towards peace, the issue of violence against women cannot be ignored. For decision-makers and programmes that have the explicit aim of addressing violence against women in the longer term, this study provides what we hope will be the beginning of a growing evidence-base to foster comprehensive, gender-informed strategies to improve the safety, health and well-being of women, men and children in conflict-affected settings.

For peer review only

Funding
This work was supported by funding from the NoVo Foundation, the Sigrid Rausing Trust and the Economic and Social Research Council (ESRC).

Competing Interests
None declared.

Author Contributions
MH, CZ, CW, LK were responsible for the design and conduct of the study. MH, DK were responsible for field management and study instrument development. MT, HL contributed to national level data collection procedures and oversight. All authors contributed to the development and adaptation of the questionnaires and interpretation of the data. MH was responsible for statistical data analysis. MH, CZ, CW drafted the manuscript which was reviewed and approved by all authors. We are grateful to all of the study respondents who participated in the study. We also gratefully acknowledge the dedicated commitment of IRC country staff and the research field staff.

Ethical approval for this study was received in 2008 from the London School of Hygiene & Tropical Medicine Ethics Committee. Local ethics approval was received from the Ministry of Family, Women and Social Affairs in Côte d'Ivoire.

For peer review only

For peer review only - http://bmjopen.bmj.com/site/about/guidelines.xhtml

References

1. Bastick, M., K. Grimm, and R. Kunz, *Sexual Violence in Armed Conflict: Global Overview and Implications for the Security Sector*. 2007, Geneva Centre for the Democratic Control of Armed Forces: Geneva. p. 216.
2. United Nations High Commissioner for Refugees (UNHCR), *Action against Sexual and Gender-Based Violence: An Updated Strategy*. 2011, UNHCR.
3. UK Department for International Development (DfID), *Press release: Helping millions of women and girls access better justice and support against violence*. 2012, DfID: London.
4. UK Foreign & Commonwealth Office and UK Department for International Development. *News Story: UK announces additional funding to address conflict sexual violence*. 2013 [cited; Available from: https://www.gov.uk/government/news/uk-announces-additional-funding-to-address-conflict-sexual-violence.
5. World Health Organization (WHO), *Meeting Report: Sexual Violence in Conflict: Data and Data Collection Methods*. 2008, WHO: Geneva.
6. Casey, S.E., et al., *Care-seeking behavior by survivors of sexual assault in the Democratic Republic of the Congo.* Am J Public Health, 2011. **101**(6): p. 1054-5.
7. Duroch, F., M. McRae, and R.F. Grais, *Description and consequences of sexual violence in Ituri province, Democratic Republic of Congo.* BMC Int Health Hum Rights, 2011. **11**: p. 5.
8. Johnson, K., et al., *Association of sexual violence and human rights violations with physical and mental health in territories of the Eastern Democratic Republic of the Congo.* JAMA, 2010. **304**(5): p. 553-62.
9. Ministère du Plan and Macro International Inc, *Enquête Démographique et de Santé, République Démocratique du Congo 2007*. 2008, Ministère du Plan, Macro International: Calverton, Maryland, USA.
10. Vinck, P. and P.N. Pham, *Association of exposure to intimate-partner physical violence and potentially traumatic war-related events with mental health in Liberia.* Soc Sci Med, 2013. **77**: p. 41-9.
11. Watts, C. and C. Zimmerman, *Violence against women: Global scope and magnitude.* Lancet, 2002. **359**: p. 1232-1237.
12. Geneva Declaration Secretariat, *Global Burden of Armed Violence 2011: Lethal Encounters*. 2011, Cambridge University Press: Cambridge.
13. Peterman, A., T. Palermo, and C. Bredenkamp, *Estimates and determinants of sexual violence against women in the Democratic Republic of Congo.* Am J Public Health, 2011. **101**(6): p. 1060-7.
14. Bartels, S.A., et al., *Sexual violence trends between 2004 and 2008 in South Kivu, Democratic Republic of Congo.* Prehosp Disaster Med, 2011. **26**(6): p. 408-13.
15. Human Security Report Project, *Human Security Report 2012: Sexual Violence, Education, and War: Beyond the Mainstream Narrative*. 2012, Vancouver: Human Security Press.
16. Bartels, S., et al., *Militarized Sexual Violence in South Kivu, Democratic Republic of Congo.* J Interpers Violence, 2012.
17. World Health Organization (WHO), *Executive Summary: A research agenda for sexual violence in humanitarian, conflict and post-conflict settings*. 2012, World Health Organization.
18. Koffi, T., *Côte d'Ivoire - l'agonie du jardin du grand reve au desastre*. 2006, Abidjan: CEDA/NEI.
19. Hellweg, J., *Hunting the Ethical State: The Benkadi Movement of Côte d'Ivoire*. 2011: University of Chicago Press.
20. McGovern, M., *Making War in Côte d'Ivoire*. 2011: C Hurst & Co Publishers Ltd.
21. Balint-Kurti, D., *Côte d'Ivoire's Force Nouvelles*, in *Chatham House: Africa Programme Armed Non-State Actors Series*. 2007, Chatham House: London.

For peer review only

22. Human Rights Watch (HRW), *"A Long Way from Reconciliation" - Abusive Military Crackdown in Response to Security Threats in Côte d'Ivoire*. 2012, Human Rights Watch: New York.
23. United Nations Development Programme (UNDP), *The conflict in Côte d'Ivoire and its effect on West African countries: A perspective from the ground*. 2011, UNDP, Regional Bureau for Africa.
24. IRIN Africa, *Analysis: The dynamics of inter-communal violence in Mali*, in *IRIN - Humanitarian news and analysis*. 2013, UN Office for the Coordination of Humanitarian Affairs.
25. Garcia-Moreno, C., et al., *Prevalence of intimate partner violence: findings from the WHO multi-country study on women's health and domestic violence.* Lancet, 2006. **368**(9543): p. 1260-9.
26. Zimmerman, C., et al., *Asylum-Seeking Women, Violence & Health: Results from a Pilot Study in Scotland and Belgium*. 2009, London School of Hygiene & Tropical Medicine (LSHTM) and Scottish Refugee Council (SRC): London.
27. Abramsky, T., et al., *A community mobilisation intervention to prevent violence against women and reduce HIV/AIDS risk in Kampala, Uganda (the SASA! Study): study protocol for a cluster randomised controlled trial.* Trials, 2012. **13**: p. 96.
28. Mollica, R.F., *Measuring Trauma, Measuring Torture*. 2004, Cambridge: Harvard Program in Refugee Trauma.
29. StataCorp, *Statistical Software: Release 11.1*. 2009, Stata Corporation: College Station, TX (USA).
30. Demographic and Health Survey, *Côte d'Ivoire 2005*, Institut National de la Statistique (INS), Editor. 2005, Measure DHS: Côte d'Ivoire.
31. Garcia-Moreno, C., et al., *WHO Multi-Country Study on Women's Health and Domestic Violence against Women*. 2005, WHO: Genva.
32. Dunkle, K.L., et al., *Perpetration of partner violence and HIV risk behaviour among young men in the rural Eastern Cape, South Africa.* AIDS, 2006. **20**(16): p. 2107-14.
33. Ahonsi, B.A., *Policy Notes: Towards More Informed Responses to Gender Violence and HIV/AIDS in Post-Conflict West African Settings*, N. Afrikainstitutet, Editor. 2010, The Nordic Africa Institute, Conflict, Displacement and Transformation: Uppsala.

For peer review only

For peer review only - http://bmjopen.bmj.com/site/about/guidelines.xhtml

Table 1. Weighted study population demographics and comparison with regional figures.

	Violence survey data		Comparative DHS data from same study regions	
**CHARACTERISTICS**	**WOMEN**	**MEN**	**WOMEN**	**MEN**
**Age Range (years)**	*%*		*%*	
15-19	23·7	22·6	24·0	23·3
20-24	20·9	16·8	19·9	20·7
25-29	17·8	15·2	16·4	13·7
30-34	14·0	15·7	12·6	13·0
35-39	10·0	13·4	10·3	11·0
40-44	8·6	9·3	9·9	9·3
45-49	5·0	7·0	6·9	9·1
Total N	*1411*	*1265*	*1407*	*1110*
**Highest educational attainment**				
Primary	19·2	24·0	30·6	31·0
Secondary	11·1	29·9	16·1	35·5
Higher	0·1	1·6	1·0	3·7
Not reported/No schooling	69·6	42·9	52·3	29·8
Total N	*1413*	*1265*	*1407*	*1110*
**Current Living & Partnership Status**				
Living with partner (married/boyfriend/girlfriend)	63·6	51·8	50·3	39·5
Not living with partner (married/boyfriend/girlfriend)	23·6	24·8	15·0	9·1
No partner reported	9·5	21·8	34·7	51·3
Not reported	3·3	1·6	0·0	0·0
Total N	*1413*	*1265*	*1407*	*1110*
**Religion**				
Catholic	13·8	11·2	17·8	18·0
Protestant / Evangelical / Other Christian	33·5	22·.1	32·5	24·9
Muslim	18·2	18·.7	22·.9	21·6
Animist (traditional)	8·6	30·2	25·2	33·8
Other / None reported	18·2	12·9	1·6	1·6
Total N	*1412*	*1265*	*1407*	*1110*
**Number of children**				
None	21·5	45·4	25·0	46·4
1-3	45·2	33·2	39·4	27·5
4-6	25·0	14·7	22·6	15·7
7-9	7·2	4·8	10·1	7·2
10-16	1·2	2·0	2·9	3·1
17+	0	0	0	0·2
Total N	*1413*	*1264*	*1407*	*1110*
**Population in conflict-affected zones**				
Rebel controlled	28·5	30·2	29·.1	31·.3
UN protected	43·9	42·9	36·9	38·7
National army controlled	27·7	26.·9	34·.0	30·0
Total N	*1413*	*1265*	*1407*	*1110*
**Traumatic conflict-related events**				
4 or less experiences	75.5	82.5	n/a	n/a
5 or more experiences	21.5	17.5		
Total N	*1412*	*1263*		

For peer review only

For peer review only - http://bmjopen.bmj.com/site/about/guidelines.xhtml

Table 2. Prevalence of interpersonal violence exposures (sexual and physical violence) by sex.

Violence type	WOMEN			MEN			p-value*
**All Violence** (any perpetrator, all respondents)							
Sexual violence	%	(95% CI)	N	%	(95% CI)	N	
Since age 15	32·9	(28·1,38·1)	1408	5·9	(3·6,9·6)	1256	0·00
After the Crisis / Last 12 months	15·1	(11·9,19·0)	1408	0·1	(0·0,0·8)	1256	0·00
Physical violence							
Since age 15	47·6	(41·9,53·4)	1413	38·0	(29·7,47·2)	1265	0·05
After the Crisis / Last 12 months	21·2	(16·0,27·6)	1413	12·2	(8·9,16·4)	1265	0.00
Physical and/or sexual							
Since age 15	57·1	(51·7,62·3)	1413	40·2[1]	(31·0,50·0)	1265	0·01
After the conflict / Last 12 months	29·9	(25·3, 34·9)	1413	12·3[1]	(8·9,16·6)	1265	0·00
**Intimate partner violence** (among ever-partnered)							
Sexual violence							
Lifetime	29·1	(22·3,36·9)	1339	n/d	n/d	n/d	n/d
After the Crisis / Last 12 months	14·9	(11·5,19·2)	1332	n/d	n/d	n/d	n/d
Physical violence (any)							
Lifetime	38·4	(31·7,45·5)	1337	19·8	(12·8,29·4)	1119	0·00
After the Crisis / Last 12 months	20·9	(15·5,27·7)	1339	9·9	(6·8,14·3)	1120	0·00
Physical violence (severe violence)							
Lifetime	23·9	(18·2,30·8)	1337	9·3	(5·9,14·4)	1119	0·00
After the Crisis / Last 12 months	11·6	(6·8, 19·1)	1339	4·2	(2·7,6·5)	1120	0·03
Physical and/or sexual							
Lifetime	49·8	(42·3,57·4)	1339	n/d	n/d	n/d	n/d
After the Crisis / Last 12 months	29·7	(24·9,35·0)	1339	n/d	n/d	n/d	n/d
**Non-partner violence** (among all respondents)							
Sexual violence							
Since age 15	9·9	(7·1,13·8)	1408	5·9	(3·6,9·6)	1256	0·03
After the Crisis / Last 12 months	1·1	(0·6,1·8)	1408	0·1	(0·0,0·8)	1256	0·01
Physical violence							
Since age 15	23·7	(18·4, 29·9)	1412	27·1	(19·9, 35·5)	1257	0·42
After the Crisis / Last 12 months	1·9	(1·3,3·2)	1412	3·6	(2·6, 4·9)	1257	0·02
Physical and/or sexual							
Since age 15	27·7	(22·1,34·0)	1413	29·9	(22·3,38·7)	1265	0·60
After the Crisis / Last 12 months	3·0	(1·8,4·8)	1413	3·6	(2·6,5·0)	1265	0·42
**Child Sexual Abuse** (among all respondents)							
Yes	7·3	(4·9,10·8)	1413	3·3	(1·7,6·5)	1265	0.04
**First sex forced** (among all respondents)							
Yes	14·8	(12·0,18·0)	1333	3·3	(2·4,4·5)	1135	0·00

* p-value denotes difference between women and men
+ Among ever-partnered women and men
n/d = data not available
[1] Does not include sexual violence by an intimate partner.
'All statistics are weighted percentages. Denominators are the sum of the survey weights in the subpopulations of women and men.

For peer review only

Table 3. Prevalence of non-partner violence perpetrator types by conflict time period and sex.

Non-partner violence perpetrators	BEFORE CRISIS (pre-1999)			DURING CRISIS (2000-2007)			AFTER CRISIS (2007-2008)			LIFETIME		
**Sexual violence (>15 years old)** ***Forced or coerced sex***	**Women (%)** (n=1408)	**Men (%)** (n=1256)	p-value	**Women (%)** (n=1408)	**Men (%)** (n=1256)	p-value	**Women (%)** (n=1408)	**Men (%)** (n=1256)	p-value	**Women (%)** (n=1408)	**Men (%)** (n=1256)	p-value
**Any perpetrator (male/female)**	**5·4**	**3·6**	**0·36**	**4·0**	**2·2**	**0·20**	**1·1**	**0·1**	**0·11**	**9·9**	**5·9**	**0·09**
Family member (male)	0·6	0·0	0·25	0·6	0·1	0·26	0·0	0·0	0·52	1·1	0·1	0·11
Family member (female)	0·1	0·2	0·48	0·1	0·2	0·46	0·0	0·0	0·52	0·1	0·5	0·35
Acquaintance (male)	1·2	0·2	0·19	0·8	0·2	0·24	0·1	0·0	0·43	2·0	0·4	0·08
Acquaintance (female)	0·2	2·2	0·02	0·1	1·3	0·06	0·0	0·3	0·08	0·3	3·4	0·00
Acquaintance (sex not reported)	0·1	0·0	0·44	0·1	0·0	0·54	0·0	0·0	0·52	0·2	0·0	0·37
Stranger/Other not identified (male)	2·6	0·0	0·02	1·9	0·1	0·03	0·2	0·0	0·38	4·5	0·1	0·00
Stranger/Other not identified (female)	0·8	1·1	0·58	0·4	0·6	0·58	0·1	0·2	0·57	1·1	1·8	0·41
Stranger/Other not identified (sex unknown)	0·0	0·0	0·52	0·1	0·0	0·45	0·0	0·0	0·52	0·1	0·0	0·45
Combatant / Uniformed Official	0·4	0·7	0·52	0·3	0·2	0·68	0·1	0·0	0·53	0·3	0·2	0·68
**Physical violence (>15 years old)** ***Physically mistreated or hit***	**Women (%)** (n=1412)	**Men (%)** (n=1265)	p-value	**Women (%)** (n=1412)	**Men (%)** (n=1265)	p-value	**Women (%)** (n=1412)	**Men (%)** (n=1265)	p-value	**Women (%)** (n=1412)	**Men (%)** (n=1265)	p-value
**Any perpetrator (male/female)**	**15·4**	**15·2**	**0·36**	**8·9**	**12·6**	**0·13**	**1·9**	**3·6**	**0·02**	**23·6**	**26·9**	**0·27**
Family member (male)	6·4	8·7	0·09	3·2	3·7	0·18	0·9	1·1	0·10	8·9	12·9	0·03
Family member (female)	8·2	2·5	0·00	4·5	1·5	0·00	0·9	1·1	0·16	8·9	3·7	0·01
Acquaintance (male)	1·6	2·4	0·23	0·7	1·4	0·15	0·2	0·8	0·04	2·1	4·4	0·09
Acquaintance (female)	0·9	0·0	0·03	0·4	0·2	0·09	0·1	0·0	0·08	1·1	0·4	0·04
Acquaintance (sex not reported)	0·3	1·7	0·01	0·0	1·2	0·01	0·0	0·6	0·01	0·3	2·9	0·00
Stranger/Other not identified (male)	1·3	2·6	0·01	0·9	1·8	0·04	0·2	0·6	0·18	2·3	4·6	0·01
Stranger/Other not identified (female)	0·2	0·2	0·10	0·0	0·2	0·05	0·1	0·6	0·07	0·2	0·3	0·08
Stranger/Other not identified (sex unknown)	0·1	0·0	0·10	0·0	0·2	0·11	0·1	0·1	0·15	0·1	0·2	0·20
Combatant / Uniformed Official	0·0	0·6	0·04	0·9	4·7	0·00	0·0	0·2	0·61	0·9	5·3	0·00

Family includes: father/mother, father/mother-in-law, and other family members; Acquaintances include: friends, family friends, neighbours, teachers, religious leaders; Strangers/ others include: strangers, individuals not identified; Combatant/uniformed official includes: someone who attacked your village, uniformed official (i.e.-police, gendarme, military), sex unspecified.

* p-value denotes difference between women and men.

‘ All statistics are weighted percentages. Denominators are the sum of the survey weights in the subpopulations of women and men.

For peer review only - http://bmjopen.bmj.com/site/about/guidelines.xhtml

Figure 1. Prevalence of traumatic experiences by type of event, timing in relation to the Crisis and by sex. (Women: n=1412; Men: n=1263)

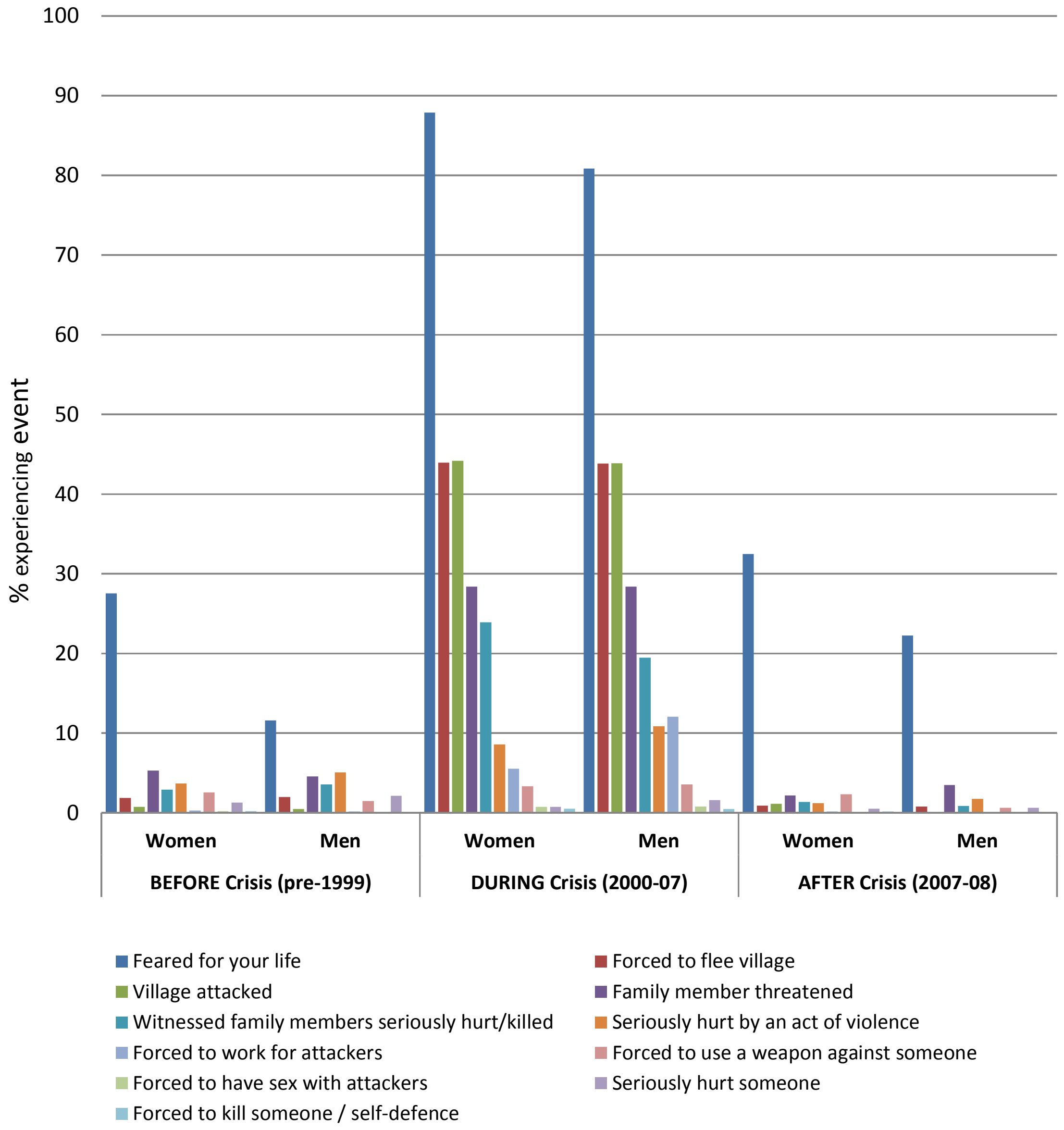

For peer review only - http://bmjopen.bmj.com/site/about/guidelines.xhtml
